# The interaction between STING and NCOA4 exacerbates lethal sepsis by orchestrating ferroptosis and inflammatory responses in macrophages

**DOI:** 10.1038/s41419-022-05115-x

**Published:** 2022-07-28

**Authors:** Jie Wu, Qinjie Liu, Xufei Zhang, Miaomiao Tan, Xuanheng Li, Peizhao Liu, Lei Wu, Fan Jiao, Zhaoyu Lin, Xiuwen Wu, Xin Wang, Yun Zhao, Jianan Ren

**Affiliations:** 1 Department of General Surgery, The Affiliated BenQ Hospital of Nanjing Medical University, Nanjing Medical University, Nanjing, China; 2 Research Institute of General Surgery, Jinling Hospital, Medical School of Nanjing University, Nanjing, China; 3 Research Institute of General Surgery, Jinling Hospital, School of Medicine, Southeast University, Nanjing, China; 4 Department of Surgery, Faculty of Medicine, The Chinese University of Hong Kong, Hong Kong, SAR China; 5 Department of Biomedical Sciences, College of Veterinary Medicine and Life Sciences, Kowloon Tong, City University of Hong Kong, Hong Kong, SAR China; 6 Shenzhen Research Institute, City University of Hong Kong, Shenzhen, China; 7 MOE Key Laboratory of Model Animals for Disease Study, Model Animal Research Center, State Key Laboratory of Pharmaceutical Biotechnology, Nanjing Drum Tower Hospital, The Affiliated Hospital of Nanjing University Medical School, Nanjing University, Nanjing, China; 8 Research Institute of General Surgery, Jinling Hospital, Nanjing Medical University, Nanjing, China

**Keywords:** Immune cell death, Bacterial infection

## Abstract

The discovery of STING-related innate immunity has recently provided a deep mechanistic understanding of immunopathy. While the detrimental effects of STING during sepsis had been well documented, the exact mechanism by which STING causes lethal sepsis remains obscure. Through single-cell RNA sequence, genetic approaches, and mass spectrometry, we demonstrate that STING promotes sepsis-induced multiple organ injury by inducing macrophage ferroptosis in a cGAS- and interferon-independent manner. Mechanistically, Q237, E316, and S322 in the CBD domain of STING are critical binding sites for the interaction with the coiled-coil domain of NCOA4. Their interaction not only triggers ferritinophagy-mediated ferroptosis, but also maintains the stability of STING dimers leading to enhanced inflammatory response, and reduces the nuclear localization of NCOA4, which impairs the transcription factor coregulator function of NCOA4. Meanwhile, we identified HET0016 by high throughput screening, a selective 20-HETE synthase inhibitor, decreased STING-induced ferroptosis in peripheral blood mononuclear cells from patients with sepsis and mortality in septic mice model. Our findings uncover a novel mechanism by which the interaction between STING and NCOA4 regulates innate immune response and ferroptosis, which can be reversed by HET0016, providing mechanistic and promising targets insights into sepsis.

## Introduction

Sepsis is a worldwide public health concern due to its high morbidity and mortality, with a global estimate of 48.9 million sepsis cases and 11.0 million sepsis-related deaths in 2017 [1]. It is re-defined as a life-threatening organ dysfunction caused by the dysregulated host response to infection by the Sepsis-3 definitions taskforce in 2016 [2]. Multiple organ dysfunction syndrome is commonly associated with poor outcome of severe sepsis. Among these, sepsis-induced intestinal injury (SII), also referred to as acute gastrointestinal injury (AGI) in the clinic, is one of the most common complications that occur during the progression of sepsis [3]. Up to 62–85% of patients in the ICU suffered SII [4]. Clinical trials have also proved that higher SII grades are significantly associated with immune disorders and a higher risk of mortality in sepsis [4–6]. Therefore, knowledge of the mechanisms underlying the interplay between SII and immune pathways is critical for understanding the pathophysiology of sepsis and developing potential new therapies.

As previously reported, both pathogen-associated molecular pattern molecules (PAMPs) from microbes and damage-associated molecular pattern molecules (DAMPs) from hosts initiate the innate immune response at the beginning of infection, which is a crucial step in developing sepsis [7]. Stimulator of interferon response cGAMP interactor 1 (STING1) is a key signaling adaptor protein that responds to PAMPs (e.g., cyclic dinucleotides [CDNs] derived from pathogens) and DAMPs (e.g., DNA and cGAMP from damaged host cells) [8]. It reported that STING plays a fundamental role in exacerbating sepsis via the production of type I interferons (IFNs) and proinflammatory cytokines [9]. In addition to this classical function, emerging evidence has implicated detrimental impacts of STING in acute or chronic inflammatory diseases by inducing various regulated cell death, such as apoptosis, necroptosis, and pyroptosis [10].

Various types of regulated cell death have also been reported in sepsis-induced organ disfunction [11–13]. Among them, ferroptosis, caused by cytoplasm iron overload-induced lipid peroxidation accompanied by destroyed antioxidation systems, has recently attracted attention in inflammatory conditions due to its extensive implications in immunity [12, 14]. Although STING has been connected to glutathione peroxidase 4 (GPX4)-related lipid peroxidation in ferroptosis, its role in regulating cytoplasm iron and ferroptosis in different cells is rarely reported. Moreover, GPX4 has been reported to have multiple divergent effects on inflammatory and immune responses and is also related to pyroptosis. Given that increased intracellular iron is the fundamental characteristic of ferroptosis, our previous clinical study showed that iron supplementation is an independent risk factor of mortality in sepsis [15]. In addition, excessive lipid peroxidation mediated by STING activation has been proved in a mouse model of intestinal ischemia-reperfusion injury [16]. These suggest that there are other unidentified mechanisms regulating STING-induced ferroptosis in sepsis. Thus, the mechanism by which STING induces ferroptosis influences ferroptosis remains to be answered.

Here, we demonstrated a unique pathway of STING-mediated ferroptosis in lethal sepsis. Mechanistically, these effects are independent of the classical downstream of STING pathway. Instead, STING drives cell death by directly interacting with cytoplasmic nuclear receptor coactivator 4 (NCOA4) to release free ferrous iron and lead to the lipid peroxidation cascade. Meanwhile, the interaction between cytoplasmic NCOA4 and STING enhances the formation of STING dimers and decreases the level of intranuclear NCOA4. We further identified a promising compound, HET0016, targeting STING-dependent ferroptosis in sepsis. Together, these findings provide a framework to understand the crosstalk between STING and ferroptosis and highlight the potential application of STING-induced ferritinophagy for the targets of immunopathy disorders, such as cancer, infectious diseases, and autoimmune diseases.

## Results

### Macrophage-specific STING1 is responsible for sepsis-related death which is lipid peroxidation relevant

**Fig. 1:**
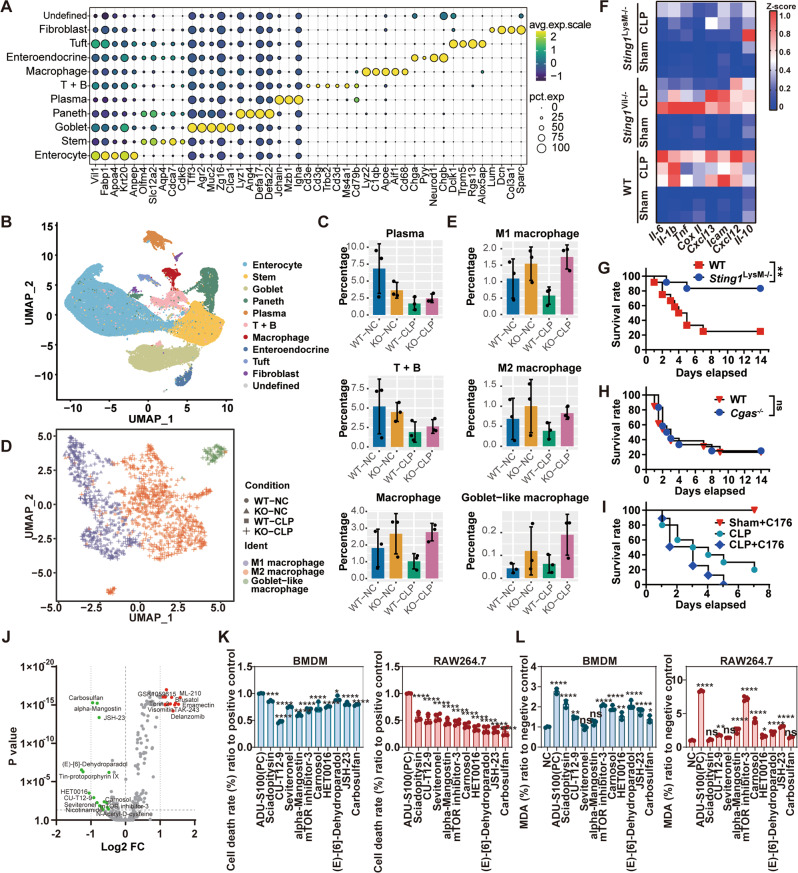
STING is responsible for macrophage lipid peroxidation and cell death. **A** Expression of indicated cell-type-specific genes is plotted across all cell types from the intestine of WT or *Sting1*^*−/−*^ mice subjected with or without CLP model (*n* = 3 per group). avg.exp.scale scaled average expression of the gene, pct.exp percentage of cells that express the gene. **B** UMAP plots displaying 62,307 single cells from the intestine of WT or *Sting1*^*−/−*^ mice subjected with or without CLP model divided into 11 subtypes, based on the expression of known marker genes (*n* = 3 per group). **C** Bar plots compare the percentages of plasma cells, T and B cells, and macrophages in WT or *Sting1*^*−/−*^ mice subjected with or without CLP model. **D** Unsupervised clustering of macrophages (*n* = 1341 cells total) identified three subclusters of macrophages. **E** Bar plots compare the percentages of M1, M2, and goblet-like macrophages in WT or *Sting1*^−*/*−^ mice subjected with or without CLP model. **F** Heatmap of inflammatory cytokine mRNA changes in intestinal tissue of indicated mice with or without sepsis model for 24 h (*n* = 3 per group). **G**–**I** Survival analysis of indicated mice is shown (*n* = 13 per group). Mice were repeated intraperitoneal administration of C-176 (13.42 mg/kg) at 2 h before CLP and 12, 24, 48, and 72 h after CLP. **J** Volcano plot of cytotoxicity fold change after treating with compounds library in ADU-S100 (29 μM) stimulated RAW264.7 cells for 24 h (*n* = 3 per group). **K**, **L** Cell death rate (**K**) and production of MDA **L** assayed from BMDMs or RAW264.7 cells treated with ADU-S100 (29 μM) in the absence or presence of indicated bioactive compounds (5 μM) for 24 h (*n* = 3 per group). Data are shown as mean ± SD, and analysis by one-way ANOVA test. CLP cecal ligation and puncture, MDA malondialdehyde, BMDMs bone marrow-derived macrophages. *P < 0.05, **P < 0.005, ***P < 0.0005, ****P < 0.0001.

To identify the cell type in which STING activation leads to tissue damage during sepsis, a mouse model of severe sepsis induced by cecal ligation and puncture (CLP) with resuscitation fluid alone without antibiotic treatment was used. Then we tested the impact of *Sting1* depletion on sepsis at the single-cell level by conducting single-cell RNA sequencing (sc-seq) analysis of cells derived from wildtype (WT) and *Sting1*^*−/−*^ intestinal tissue from sham or CLP mice. We profiled 62,307 individual cells and identified 11 individual clusters of cell types that we confirmed to express cell lineage marker genes (Fig. 1A, B).

Enrichment of cell clusters was calculated (Supplementary Fig. S1A). The distributions of WT and *Sting1*^*−/−*^ cells, especially those involved in the immune response during sepsis, differed between the clusters (Fig. 1C and Supplementary Fig. S1B). Overall, the proportion of immune cells was higher in *Sting1*^*−/−*^ mice than in WT mice after CLP. Notably, macrophages were markedly decreased in WT mice with sepsis, while their percentages were higher both in *Sting1*^−*/−*^ mice with or without CLP modeling (Fig. 1C). Based on several published articles [17–19], we further divided the macrophage clusters into three subtypes: M1(marked as *Itgax* and *Ccr2*), M2 (marked as *Mertk* and *Mrc1*), and goblet-like macrophages (marker as *Agr2* or *Muc2*,) (Fig. 1D, E and Supplementary Fig. S1C). Interestingly, the proportions of M1 and M2 macrophages were decreased in CLP-induced septic WT mice but not in the *Sting1*^−*/−*^ mice (Fig. 1E). To evaluate the role of STING in macrophages, we generated the conditional ablation of *Sting1* in macrophages (*Sting1*^*LysM−/−*^) or intestinal epithelial cells (*Sting1*^*Vil−/−*^) in mice by using Cre/loxP technology. The results revealed that STING in macrophages but not in intestinal epithelial cells plays a major role in mediating septic death and excessive inflammation (Fig. 1F, G and Supplementary Fig. S1E). Moreover, transmission electron microscope (TEM) revealed that the cells in the lamina propria appeared to be more severely injured than those in the epithelial layer in WT mice with sepsis (Supplementary Fig. S1D). These results prompt that the STING in macrophages was crucial for the development of sepsis.

Considering that STING activation by the DNA sensor cyclic GMP-AMP (cGAMP) synthase (cGAS) in response to DNA plays an indispensable role in the immune response [20], we generated *Cgas* knockout mice. The survival analysis showed that *Cgas* knockout failed to protect against septic death (Fig. 1H) and decrease the production of *Ifnb1* (Supplementary Fig. S1F). Meanwhile, C-176, a covalent mouse STING inhibitor by inhibiting palmitoylation at C88/91 of STING, also failed to significantly improve the survival rate of mice with sepsis (Fig. 1I). Therefore, these findings demonstrate that death from sepsis caused by STING is in part mediated by IFN-independent activities.

Thus, to explore the potential mechanism, we screened a compound library, containing 625 compounds that target lipid peroxidation, autophagy, cell death, mitochondrial metabolism, etc., redox-related pathways. The effect of compounds on cell activity was estimated by lactate dehydrogenase (LDH) detection kit in RAW264.7 cells stimulated with ADU-S100, a STING activator (Fig. 1J). We selected the top fifteen compounds that promoted STING-induced cell death and the top fifteen that inhibited STING-induced cell death for further lipid reactive oxygen species (ROS) production analysis (Supplementary Fig. S1G). Then, the top ten compounds that inhibited STING-induced lipid ROS production were chosen (Supplementary Fig. S1H). Their ability in inhibiting cell death and malondialdehyde (MDA), a product of lipid peroxidation, after STING activation was examined in bone marrow-derived macrophages (BMDMs) and RAW264.7 cells (Fig. 1K, L). The results showed that anti-lipid peroxidation (NF-E2-related factor 2 [NRF2] activators, lipid peroxidase inhibitors) and anti-inflammation (NF-κB inhibitors) were the major targets, followed by anti-steroid biosynthesis (CYP17 lyase and CYP3A4 inhibitors) and isocitrate dehydrogenase inhibitor. Collectively, these findings demonstrate that macrophage-specific STING1 is responsible for cell death that is lipid peroxidation-dependent.

### Activation of STING in macrophages is essential for inducing ferroptotic injury in lethal sepsis

**Fig. 2:**
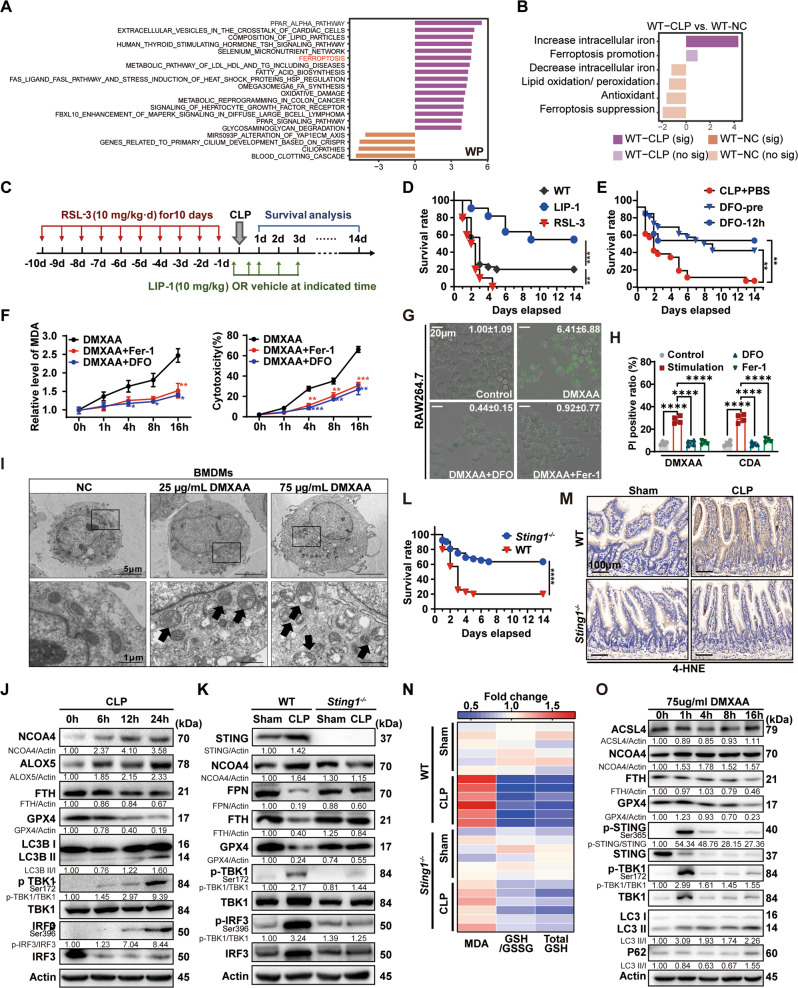
STING-mediated ferroptosis in macrophages plays an essential role in lethal sepsis. **A** Differential pathways enriched in WT and *Sting1*^*−/−*^ mice after CLP model by GSVA. **B** Ferroptosis-related GSVA enrichment score under different conditions. Sig significantly enriched, no sig not significantly enriched. **C** Schematic diagram showing the animal modeling experimental procedures. **D** Survival analysis of the indicated mice in CLP-induced sepsis with or without treatment of LIP-1 (10 mg/kg) or RSL3 (10 mg/kg) (*n* = 20 per group). **E** Survival analysis of the indicated mice is shown. Mice were treated with or without DFO (100 mg/kg) at 2 h before CLP and 12, 24, 48, and 72 h after CLP in DFO-pre group. Mice were treated with or without DFO (100 mg/kg) at 12, 24, 48, and 72 h after CLP in DFO-12 h group (*n* = 13 per group). **F** Relative production of MDA and LDH release in BMDMs stimulated with DMXAA (75 μg/mL) in the absence or presence of Ferrostatin-1 (Fer-1, 10 µM) or deferoxamine (DFO, 100 nM) at indicated time points (*n* = 3 per group). **G** Live-cell fluorescence imaging of BMDMs with Liperfluo (10 μM). BMDMs were stimulated with DMXAA (75 μg/mL) in the absence or presence of Fer-1 (10 µM) or DFO (100 nM) at 16 h. Typical changes in fluorescence are shown (*n* = 3 per group). The fold change of integrated density in each group was indicated in the upper-right corner. **H** Quantification of cell death assessed by propidium iodide (5 μg/mL) staining (*n* = 4 per group). **I** Representative transmission electron microscopy (TEM) characterization of ferroptosis in BMDMs treated with 25 μg/mL or 75 μg/mL DMXAA for 16 h. The black arrows indicate the typical morphological characteristics of ferroptosis. The lower panel of each treatment shows the high magnification image of the upper panel. Scale bars are displayed in each image. **J** Immunoblot analysis of STING and ferroptosis pathway in the intestinal tissue of WT mice subjected with or without CLP at representative time points. **K** Immunoblot analysis of STING and ferroptosis pathway in the intestinal tissue of WT or *Sting1*^*−/−*^ mice subjected with or without CLP at 24 h. **L** Survival analysis of the indicated mice in CLP-induced sepsis (*n* = 13 per group). **M** Representative immunohistochemical images of 4-HNE in intestinal tissues of WT or *Sting1*^*−/−*^ mice subjected with or without CLP at 24 h. Scale bars are displayed in each image. **N** Heatmap of MDA, GSH/GSSG, and total GSH changes in intestinal tissue of WT or *Sting1*^*−/−*^ mice subjected with or without sepsis model for 24 h (*n* = 6 per group). **O** RAW264.7 cells treated with 75 μg/mL DMXAA at different time points. Ferroptosis and STING pathway were assessed by immunoblot. Data are shown as mean ± SD. Data are analyzed by two-sided moderated *t*-tests using limma (**A**, **B**), one-way ANOVA test (**F**, **H**, **N**). GSVA gene set variation analysis, CLP cecal ligation and puncture, LIP-1 liproxstatin-1, DFO deferoxamine, CDA c-di-AMP, MDA malondialdehyde, BMDMs bone marrow-derived macrophages, ANOVA analysis of variance. *P < 0.05, **P < 0.005, ***P < 0.0005, ****P < 0.0001.

To dissect the further molecular mechanism by which STING induced lipid peroxidation in macrophages, we performed gene set variation analysis (GSVA) of the WikiPathway database in all the macrophage clusters obtained in sc-seq analysis of WT and *Sting1*^*−/−*^ mice described above. It showed that ferroptosis is one of the top pathways enriched in the macrophage clusters of *Sting1*^*−/−*^ CLP mice (Fig. 2A). Thus, we next collected gene sets and classified ferroptosis-related genes into six subgroups: increase intracellular iron, promote ferroptosis, increase lipid oxidation/peroxidation, inhibit oxidation, suppress ferroptosis, or decrease intracellular iron, according to previous studies and database shown in “Method”. The details of the gene sets and their networks are shown in Supplementary Table S1 and Supplementary Fig. S2A, B. The GSVA of ferroptosis gene sets highlight the critical role of STING in increasing intracellular iron levels and promoting ferroptosis (Fig. 2B and Supplementary Fig. S2B).

Then we built a mouse model of severe sepsis treated with liproxstatin-1 (LIP-1), a ferroptosis inhibitor; or RSL3, a ferroptosis inducer, to test whether the ferroptosis was pivotal in septic mice (Fig. 2C). Survival analysis showed that RSL3 pretreatment significantly increased mortality, whereas LIP-1 treatment decreased the mortality of septic mice compared to the untreated CLP mice (Fig. 2D). Moreover, to evaluate the utility of iron chelation in treating sepsis, we divided CLP mice into three treatment regimens: phosphate-buffered saline (PBS) control, deferoxamine (DFO, an iron chelation agent) pretreatment (receiving DFO before CLP), and DFO posttreatment (receiving DFO at 12 h after CLP). Survival analysis showed that treatment of DFO significantly improved the survival rates compared to the PBS injection group, regardless of the time of administration (Fig. 2E). Similarly, the degree of organs damage (Supplementary Fig. S2C, D), intestinal barrier injury (Supplementary Fig. S2E), production of systemic and intestinal inflammatory cytokines (Supplementary Fig. S2F, G) was increased in CLP alone group, which could be reversed by LIP-1 treatment and exacerbated by RSL3 administration. Moreover, western blot (Supplementary Fig. S3A) and immunohistochemistry (IHC) (Supplementary Fig. S3B, C) analyses revealed that ferroptosis-related proteins and STING pathway-related proteins were changed more significantly in the CLP group than in sham control. These data suggest that ferroptosis and STING activation are common features in sepsis.

Next, we sought to determine whether STING activation in macrophages can trigger ferroptosis. We conducted a timed analysis of DMXAA, di-ABZI, or c-di-AMP sodium (CDA), both are the specific agonists of STING, treatment of BMDMs and RAW264.7 cells to assess the relationship between STING activation and ferroptosis occurrence. Our results showed that the levels of MDA production and cytotoxicity (Fig. 2F) changed significantly at 16 h. Consistently, the rate of cell death and levels of lipid ROS were higher in BMDMs and RAW264.7 cells after stimulating with STING agonists, and were reversible by treatment with ferroptosis inhibitors, Fer-1 and DFO (Fig. 2G, H and Supplementary Fig. S3D, E). Then, we examined ferroptosis of BMDMs isolated from WT mice in response to treatment with DMXAA by electron microscopy. The results showed that significant microstructure changes after the stimulation, exhibited mitochondrial vacuole formation with an increased mitochondrial membrane density and the disappearance of mitochondrial cristae, which are typical morphological characteristics of ferroptosis [14] (Fig. 2I). These data show that activation of the STING pathway is involved in macrophage ferroptosis.

To clarify whether STING is the trigger of ferroptotic injury in sepsis-induced organ damage, we evaluated the activation of STING-related proteins, ferroptosis-related indexes, and mortality in *Sting1*^*−/−*^ and WT mice who suffered from CLP modeling. The results showed that phosphorylation of TANK-binding kinase 1 (TBK1), the downstream event of STING signaling, was observed at 6 h after modeling, while the consumption of GPX4, an anti-lipid peroxidation protein, and ferritin heavy chain (FTH), a subunit of ferritin, was only obvious at 12 h (Fig. 2J), the lower levels of these two indicators mean more ferroptosis occurrence. Furthermore, *Sting1* deletion in mice recovered the decrease of ferroportin (FPN), FTH, and GPX4, which are utilized as a signal of ferroptosis (Fig. 2K), improved the survival rate (Fig. 2L), and mitigated lipid peroxidation (Fig. 2M, N). In DMXAA-treated RAW264.7 cells, ferroptosis-related proteins changed significantly at 16 h, while STING activation occurred as early as one hour after stimulation (Fig. 2O). These results indicated that STING activation is a prerequisite for ferroptosis initiation.

### STING-induced ferroptosis relies on NCOA4-mediated ferritinophagy independent of TBK1

**Fig. 3:**
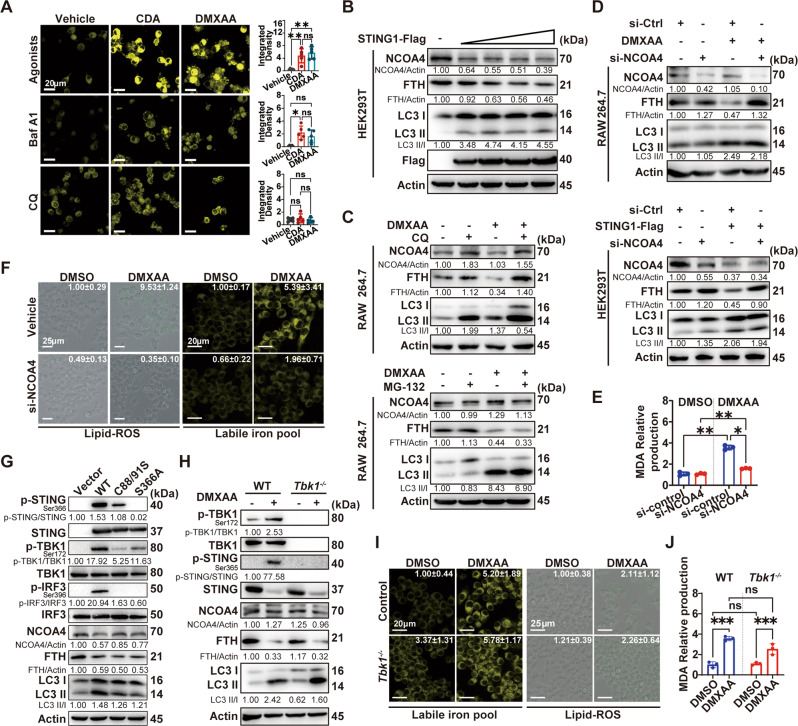
STING induces ferroptosis via NCOA4-mediated ferritinophagy is independent of TBK1. **A** FerroOrange (1 μM) staining for intracellular Fe^2+^ in RAW264.7 cells treated with indicated STING agonists in the presence or absence of Bafilomycin A1 (Baf A1, 50 nM) or Chloroquine (CQ, 10 μM) for 16 h. Typical changes in fluorescence are shown. The scale bar represents 20 μm. The integrated density in each group was indicated on the right panel. **B** Immunoblot analysis of the indicated proteins in HEK293T cells transfected with increasing concentration of *STING1*-Flag plasmid (0.25–1.50 μg). **C** Immunoblot analysis of the indicated proteins in RAW264.7 cells treated with 75 μg/mL DMXAA in the presence or absence of CQ (10 μM) or MG132 (1 μM) for 16 h. **D** Immunoblot analysis of the indicated proteins in RAW264.7 or HEK293T cells transfected with siNCOA4 (50 nM) and treated with 75 μg/mL DMXAA for 16 h or transfected with *STING1*-Flag plasmid (1.50 μg) for 24 h. **E** Relative production of MDA in RAW264.7 cells transfected with siNCOA4 (50 nM) for 36 h and treated with 75 μg/mL DMXAA for 16 h (*n* = 3 per group). **F** Live-cell fluorescence imaging of RAW264.7 cells transfected with siNCOA4 (50 nM) and treated with 75 μg/mL DMXAA for 16 h. The fold change of integrated density in each group was indicated in the upper-right corner. **G** Immunoblot analysis of the indicated proteins in HEK293T cells transfected with STING1 mutant plasmids (1.50 μg) for 24 h. **H**–**J** Immunoblot analysis of the indicated proteins (**H**), live-cell fluorescence imaging of labile iron pool or lipid ROS (**I**), and relative production of MDA (**J**) in *Tbk1*^*−/−*^ RAW264.7 cells treated with 75 μg/mL DMXAA for 16 h (*n* = 3 per group). Scale bars are displayed in each image. The fluorescence of Liperfluo was registered using an inverted EVOS™ M5000 fluorescent microscope. The fluorescence of the labile iron pool was registered using Zeiss confocal laser scanning microscopy, and the fold change of integrated density in each group was indicated at the upper-right corner (**A**, **F**, and **I**). Data are shown as mean ± SD, and analysis by two-way ANOVA test (**A**, **E**, and **J**). *P < 0.05, **P < 0.005, ***P < 0.0005, ****P < 0.0001.

Increases in the levels of intracellular free iron play a significant role in triggering ferroptosis, but it has not been evaluated in the context of STING activation. Therefore, we evaluated changes in cytoplasmic free iron levels in living cells in response to treatment with two STING agonists: CDA and DMXAA. These agonists robustly increased the level of cytoplasmic free iron in RAW264.7 cells (Fig. 3A) and BMDMs (Supplementary Fig. S4A), which suggests that STING activation plays a direct role in free iron production.

Given that NCOA4-mediated ferritinophagy has been recognized as an indispensable process to release free iron into the labile iron pool [21], we sought to determine whether NCOA4 is involved in STING-induced free iron production. RAW264.7 cells were treated with autophagosomal degradation, bafilomycin A1 (Baf A1), or chloroquine (CQ), and found that free iron levels were strongly decreased by the treatments (Fig. 3A and Supplementary Fig. S4A). Consistently, NCOA4 and FTH protein levels were decreased in HEK293T or HeLa cells transfected with the *STING1*-Flag plasmid (Fig. 3B and Supplementary Fig. S4B). These data suggest that intracellular free iron accumulation after STING activation is closely related to NCOA4-mediated ferritin degradation.

However, ubiquitin-proteasome system (UPS) also plays a complex role in regulating the STING pathway and NCOA4 degradation [22–25]. To investigate whether UPS also plays a role in STING-induced NCOA4 degradation, RAW264.7, HeLa, and HEK293T cells were pretreated with the autophagosome-lysosomal inhibitor (CQ) or the proteasome inhibitor (MG132) and then treated with or without DMXAA. We found that upon DMXAA stimulation in RAW264.7 cells (Fig. 3C) or *Sting1*-overexpressing in HeLa cells (Supplementary Fig. S4C) and HEK293T (Supplementary Fig. S4D), CQ, but not MG132, effectively restored NCOA4 and FTH protein levels without affecting their mRNA expression levels (Supplementary Fig. S4E). These observations suggest that STING induced the intracellular accumulation of free iron via the autophagy degradation system other than UPS.

To corroborate these findings, we knocked down NCOA4 expression by siRNA transfection (Supplementary Fig. S4F–I) and found that FTH expression in both RAW264.7 and HEK293T cells was restored without affecting autophagic flux (Fig. 3D). Moreover, the levels of MDA (Fig. 3E) and lipid ROS production, as well as free iron accumulation (Fig. 3F), were decreased in NCOA4 siRNA-transfected cells even after DMXAA-mediated stimulation of STING. Together, these results show that the STING pathway triggers macrophages ferroptosis through NCOA4-mediated ferritinophagy.

One of the major downstream of STING pathway is TBK1 activation-induced cytokines production [26]. Moreover, it has been reported that TBK1 also plays a role in inducing autophagy. Thus, we sought to define whether TBK1 and its downstream get involved in STING-mediated ferroptosis. We first transfected HEK293T cells with plasmids expressing STING C88/91R, which led to STING palmitoylation deficiency, or S366A, with abolished interferon regulating factor 3 (IRF3) phosphorylation [27]. Immunoblot analysis suggested that although the activation of STING and its downstream, phosphorylation of TBK1 and IRF3, was abolished, the levels of NCOA4 and FTH were still decreased after transfecting the STING1 point mutant plasmids (Fig. 3G). Furthermore, we showed that knockout of *Tbk1* in RAW264.7 cells predominantly blocked STING phosphorylation (Fig. 3H), partially decreased cell death (Supplementary Fig. S4J), suppressed production of *Ifnb* and interleukin (*Il)-6* (Supplementary Fig. S4K, L), but it failed to restrain intracellular free iron accumulation, reduction lipid ROS production (Fig. 3I), decrease level of MDA (Fig. 3J), or restore the protein levels of FTH and NCOA4 (Fig. 3H) even though their mRNA levels were higher in *Tbk1*^−*/−*^ cells than in control cells (Supplementary Fig. S4M, N). Together, these findings indicate that STING-mediated ferroptosis requires the participation of NCOA4 but not TBK1 downstream signaling.

### The CDN-binding domain of STING and coiled-coil domain of NCOA4 are required for the formation of a complex

**Fig. 4:**
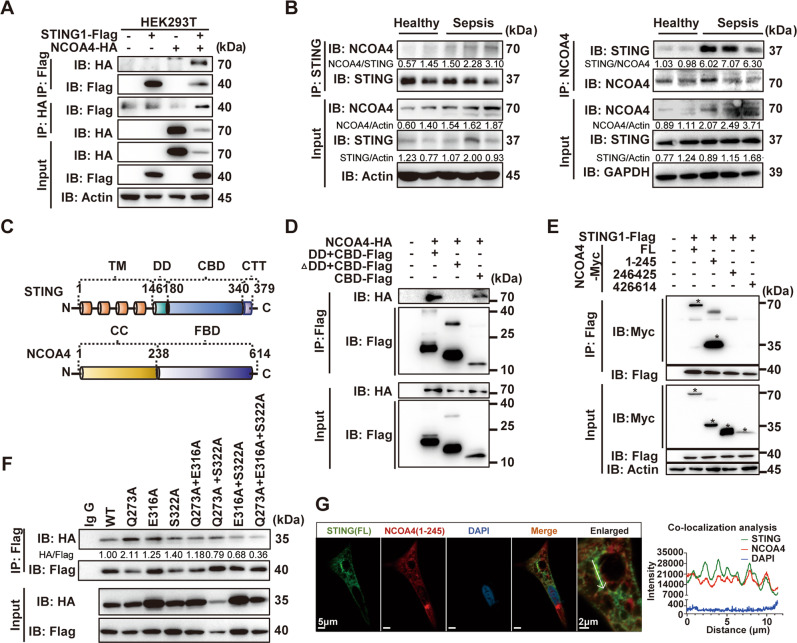
The CDN-binding domain of STING interacts with the CC domain of NCOA4. **A** Coimmunoprecipitation (Co-IP) of NCOA4 with STING in HEK293T cells. **B** Immunoprecipitation of anti-STING and anti-NCOA4 in PBMCs from healthy control and patients with sepsis. **C** Schematic structure of STING and NCOA4 protein. **D** Flag IP from lysates of HEK293T cells overexpressing Flag-tagged STING fragments and HA-tagged NCOA4 were immunoblotted. **E** Myc IP from lysates of HEK293T cells overexpressing Myc-tagged NCOA4 fragments and Flag-tagged STING1 were immunoblotted for detection of NCOA4. * Target bands. **F** Flag IP from lysates of HEK293T cells overexpressing Flag-tagged STING1 mutant plasmids and HA-tagged 1–245 NCOA4 fragments were immunoblotted. **G** Representative images of co-localization with STING (green) and 1–245 NCOA4 (red) fluorescent proteins were captured by laser scanning confocal microscopy. Enlarged images showed the confocal microscopy images of STING and NCOA4. All immunoblots are representative of at least three independent experiments with similar results. CBD CDN-binding domain, DD dimerization domain, CTT cytoplasmic-terminal tail, CC coiled-coil domain, FBD ferritin-binding domain, FL full-length, IP immunoprecipitation.

To further explore the mechanisms by which STING regulates NCOA4-mediated ferritinophagy, we used a coimmunoprecipitation assay of Flag-tagged STING and HA-tagged NCOA4 expressed in HEK293T cells. We found that STING coimmunoprecipitated with NCOA4 regardless of whether a Flag-tagged or HA-tagged antibody was used (Fig. 4A). This association was confirmed by immunoprecipitation of endogenous STING from RAW264.7 cells stimulated with CDA, which resulted in the coimmunoprecipitation of NCOA4 protein (Supplementary Fig. S5A). Subsequent coimmunoprecipitation and mass spectrometry (MS) assays showed that the STING-NCOA4 interaction also occurred in peripheral blood mononuclear cells (PBMCs) from patients with sepsis but not in those from healthy subjects (Fig. 4B and Supplementary Fig. S5B). Similarly, confocal microscopy analysis showed a strong punctate structure and co-localization between STING and NCOA4, as well as ferritin, in HEK293T cells transfected with plasmids expressing these proteins (Supplementary Fig. S5C).

The STING protein contains N-terminal transmembrane (TM) domains and a C-terminal cytoplasmic domain consisting of three domains: the dimerization domain (DD), the CDN-binding domain (CBD), and the cytoplasmic-terminal tail (CTT) domain (Fig. 4C). NCOA4 is expressed as multiple splice variants in humans, among which two isoforms are the most common: NCOA4α (614 Aa, 70 kDa) and the splice isoform NCOA4β (286 Aa, 35 kDa) (Supplementary Fig. S5D) [28]. These isoforms share the N-terminal coiled-coil (CC) domain and a small portion of the C-terminus. In addition, NCOA4α contains a C-terminal domain known as ferritin-binding domain (FBD) (Supplementary Fig. S5D). To identify the domains that mediate the STING and NCOA4 interaction, we generated mutant STING constructs lacking the TM, CBD+DD, or CTT domain (Fig. 4D and Supplementary Fig. S5E) and assessed the interactions by coimmunoprecipitation of Flag-tagged full-length (FL) or truncated STING proteins with FL NCOA4-Myc in HEK293T cells. The results showed that the DD and CBD of STING are the main domains involved in the NCOA4 interaction (Supplementary Fig. S5E). We further generated fragment of NCOA4 constructs containing 1–245 amino acid, 246–425 amino acids, or 426–614 amino acids. Coimmunoprecipitation assay confirmed the CC fragment of NCOA4 (1–245 amino acids) and the CBD of STING (180–340 amino acids) is required for the complex formation (Fig. 4D, E). Next, Discovery Studio 2019 (BIOVIA) was used to visualize the predicted contact interface and H-bonds between the two proteins (Supplementary Fig. S5F–H). To validate the predicted sites of H-bonds, we constructed mutant plasmids and performed a coimmunoprecipitation assay. The results showed that E316 and S322 were more important for interaction followed by Q273 (Fig. 4F). Moreover, confocal microscopy analysis revealed a more significant co-localization between STING and CC fragment of NCOA4 in Hela cells (Fig. 4G). Collectively, these results suggest that the CBD of STING plays an essential role in forming a complex with NCOA4 to induce ferritinophagy.

### NCOA4 interacts with STING to enable STING dimerization and decreases NCOA4 nuclear localization

**Fig. 5:**
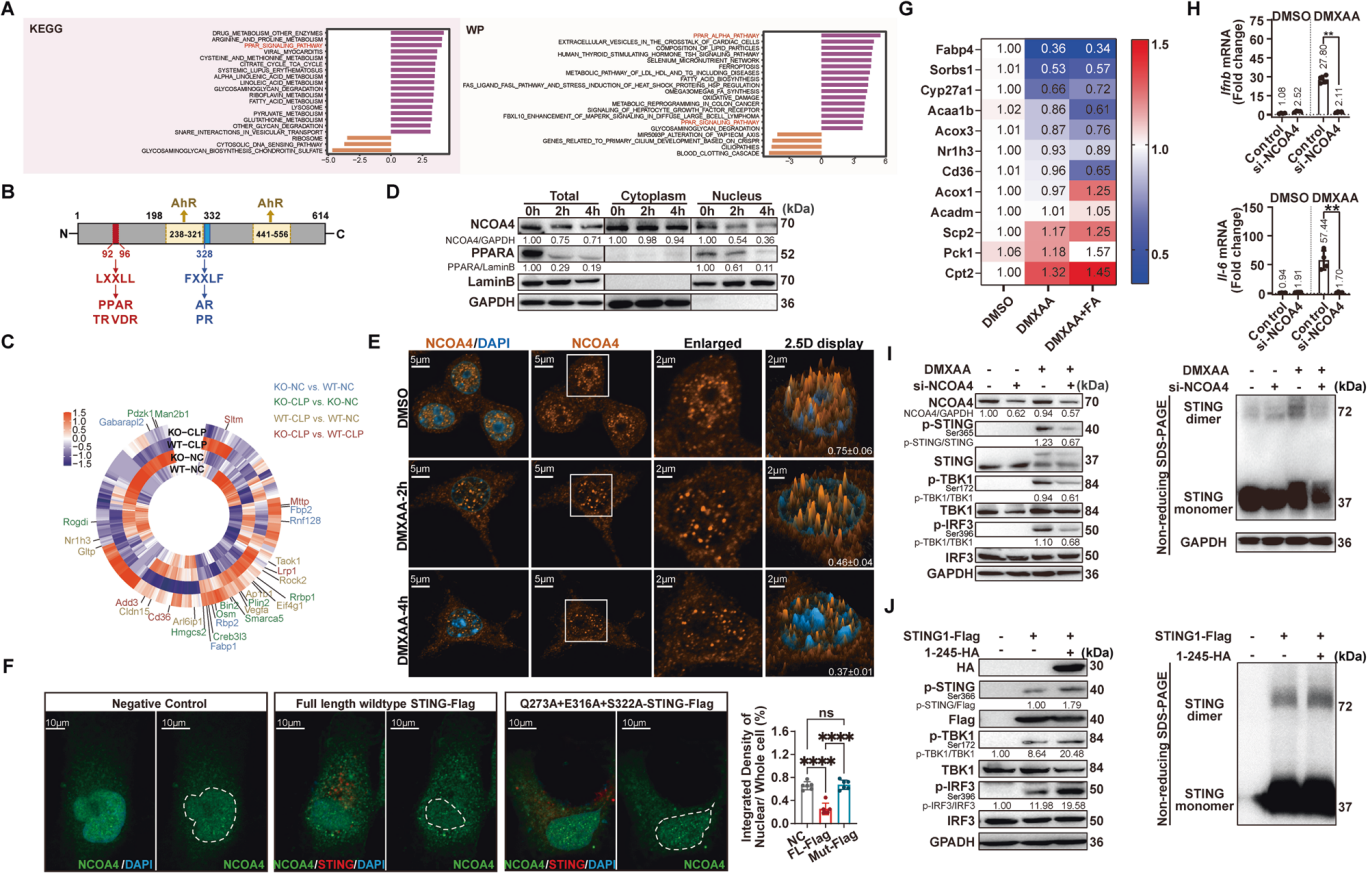
NCOA4 binds with STING to maintain STING activation that promotes ferritinophagy and lessens nuclear localization. **A** Differential pathways enriched in WT and *Sting1*^*−/−*^ mice after CLP model by GSVA. *P* values were calculated by two-sided moderated *t*-tests using limma. **B** Schematic representation of human NCOA4. The full-length NCOA4 is known as NCOA4α. The LXXLL motif located at amino acids 92–96 and the FXXLF motif located at amino acids 328–332, which is involved in interaction with PPAR, thyroid hormone (TR), vitamin D receptor (VDR), androgen receptor (AR), and progesterone receptor (PR), respectively. Amino acids 231–321 and 441–556 are domains for aryl hydrocarbon receptor (AhR) coactivation. **C** Circos plot illustrates the relative expression level of peroxisome proliferator-activated (PPAR) downstream target genes in macrophages in WT and *Sting1*^*−/−*^ mice subjected with and without CLP model. Differently expressed genes (DEGs) between indicated groups were highlighted in different colors. **D** Immunoblot analysis of the indicated proteins in RAW264.7 cells treated with 50 μg/mL DMXAA for 2 and 4 h. **E** The fluorescence distribution of NCOA4 in nucleus and cytosol after stimulated with 50 μg/mL DMXAA for 2 and 4 h (*n* = 3 per group). The peak value of the *z*-axis in the 2.5D display column represents the fluorescence intensity. The quantified nuclear localization proportion of Ncoa4 is shown in the lower right corner. **F** Hela cells were transfected with the indicated STING1 plasmids (1.5 μg) alone for 24 h. The quantitative analysis of nuclear localization of NCOA4 is shown in the rightmost panel; Green fluorescence, NCOA4; Red fluorescence, *STING1*-Flag; Blue fluorescence, DAPI; White dashed line indicated the nuclei borders. **G** Heatmap of selected PPARα target genes expression analysis by qPCR of the RAW264.7 cells after DMXAA (75 μg/mL) stimulation in combination with Fenofibric acid (FA, 100 μM) or vehicle (DMSO) for 16 h (*n* = 3 per group). **H** qPCR analysis of *Il-6* and *Ifnb* mRNA in RAW264.7 cells transfected with siNCOA4 (50 nM) and treated with 75 μg/mL DMXAA for 16 h (*n* = 4 per group). **I** Immunoblot analysis of STING pathway in RAW264.7 cells transfected with siNCOA4 and treated with 25 μg/mL DMXAA for 1 h. Non-reducing SDS–PAGE analysis of STING dimerization in indicated groups. **J** Immunoblot analysis of STING pathway in HEK293T cells transfected with an indicated plasmid (1 μg) for 24 h. Non-reducing SDS–PAGE analysis of STING dimerization in indicated groups. Western blot data show at least representative triplicates from two independent experiments with similar results (**D**, **I**, **J**) unless indicated. Data are shown as mean ± SD, and analysis by two-way ANOVA test (**F** and **H**). GSVA gene set variation analysis, GSEA gene set enrichment analysis, TFs transcription factors, CLP cecal ligation and puncture, ANOVA analysis of variance. *P < 0.05, **P < 0.005, ***P < 0.0005, ****P < 0.0001.

To better understand the molecular biological effects of the interaction between STING and NCOA4, we performed GSVA analysis of all the differentially expressed genes in macrophages of *Sting1*^*−/−*^ CLP mice with the Kyoto Encyclopedia of Genes and Genomes (KEGG), Reactome, Biocarta, and WikiPathway databases. Interestingly, we found that one of the top pathways enriched in all the databases was the peroxisome proliferator-activated receptor α (PPARα) pathway, which is closely related to lipid synthesis/metabolism and inflammation-modulating pathways (Fig. 5A and Supplementary Figs. S5I and S6A). Not only that, NCOA4 was initially discovered as a coactivator of various nuclear receptors, including androgen receptor, glucocorticoid receptor, peroxisome proliferator-activated receptor (PPAR)αγ, and aryl hydrocarbon receptor [29] (Fig. 5B). Thus, we speculated that the formation of the STING1-NCOA4 complex decreases the nuclear localization of NCOA4 and disrupts its coactivator function via cytoplasmic retention of NCOA4. To verify this, gene set enrichment analysis (GSEA) of NCOA4-related transcription factors (TFs) and a heatmap of genes downstream of these TFs revealed the significantly increased expression of PPAR and AR target genes in *Sting1*^*−/−*^ CLP mice when compared to WT modeled mice (Fig. 5C and Supplementary Fig. S6B). In addition, we evaluated changes in the NCOA4 levels in the RAW264.7 cells cytoplasm and nucleus by immunoblotting of cells fractions and immunofluorescence analysis. The results showed that after stimulation of RAW264.7 cells with DMXAA, the nuclear localization of NCOA4 was decreased (Fig. 5D, E). Consistent with this finding, the proportion of nuclear NCOA4 decreased after full-length *STING1*-Flag plasmid transfection, which was reversed by transfecting with mutation *STING1*-Flag plasmid, which the ability of interaction with NCOA4 was abolished (Fig. 5F). Furthermore, we observed that the expression of the majority of selected PPAR target genes was decreased in DMXAA-stimulated RAW264.7 cells (Fig. 5G). Together with the results of our bioinformatics analysis, these data suggest that STING physically interacts with NCOA4, affecting the nuclear localization of NCOA4 and its function in regulating PPARα transcriptional activity.

Then, we further explored whether NCOA4 binding can affect STING activation by knocking down NCOA4 in RAW264.7 cells. Intriguingly, the results showed decreased *Il-6* and *Ifnb* mRNA expression in the siNCOA4 group compared to the control group after DMXAA stimulation, indicating a positive role of NCOA4 in facilitating STING signaling (Fig. 5H). Given that the CBD of STING is required for binding to the CC domain of NCOA4α/β, we speculated that the CC domain of NCOA4 plays a functional role in STING pathway activation by stabilizing STING dimerization. The results showed that STING dimerization took place 1 h after DMXAA stimulation in control RAW264.7 cells but that this activation was weakened in the siNCOA4 group (Fig. 5I). Moreover, the dimerization of STING, as well as phosphorylation of TBK1 and IRF3, were enhanced in HEK293T cells after transfecting with the plasmid containing 1–245 amino acids of NCOA4 (Fig. 5J).

Collectively, these data support the notion of a functional interaction between the STING and NCOA4 proteins, wherein NCOA4 enhances STING dimerization and promotes STING downstream signaling while STING activation lessens the protective effects of NCOA4 as a nuclear coactivator to upregulate the downstream genes expression of PPAR.

### HET0016 protects against sepsis by hampering STING-induced ferroptosis

**Fig. 6:**
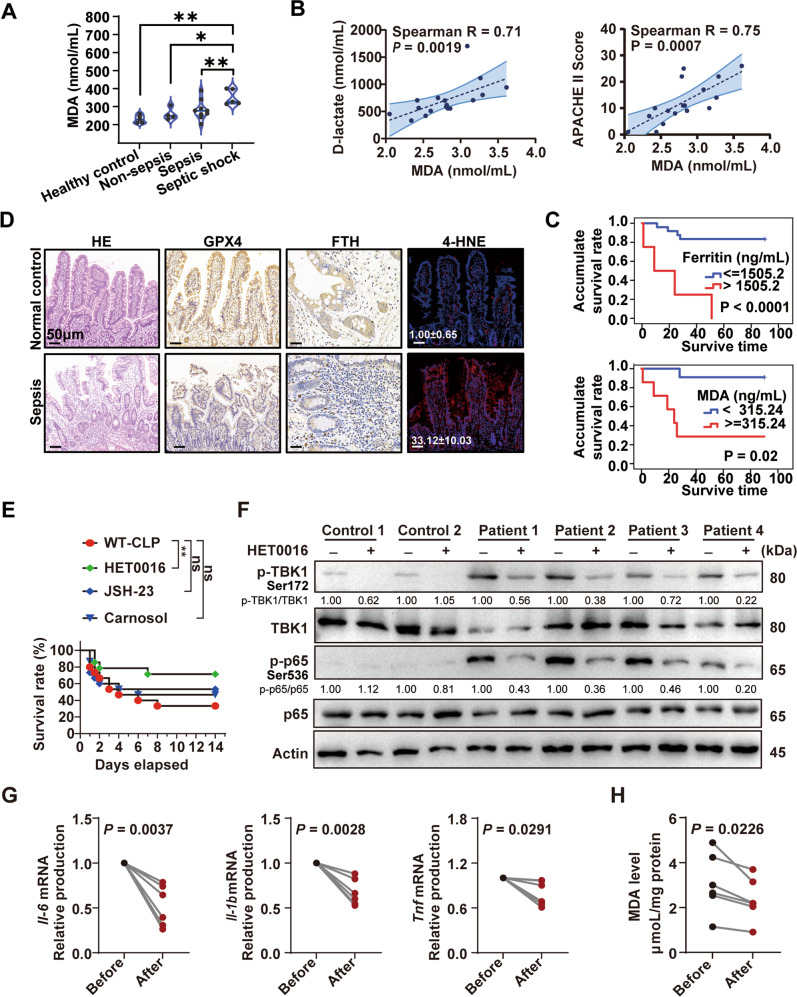
HET0016 as a lipid peroxidation inhibition protects against sepsis. **A** Levels of plasma MDA from healthy control or patients. **B** Spearman correlation analysis for plasma MDA and D-lactate or APACHE II score in all patients (*n* = 28). **C** Kaplan–Meier analysis according to a cutoff value calculated from ROC analysis. **D** Representative histopathological section images, immunohistochemical or fluorescent images in the intestinal tissues of patients with sepsis. The scale bar represents 50 μm. The quantified fluorescence intensity of 4-HNE was shown in the lower left corner. **E** Survival analysis of the indicated mice in CLP-induced sepsis with or without treatment of 10 mg/kg HET0016, 20 mg/kg JSH-23, or 10 mg/kg Carnosol at 2 h before CLP and 12, 24, 48, and 72 h after CLP (*n* = 15 per group). **F** Immunoblot analysis of p-TBK1, TBK1, p-P65, and P65 in PBMCs from patients with sepsis or healthy control, supplemented with or without HET0016 (5 μM) for 24 h (*n* = 4 per group). **G** qPCR analysis of *Il-6*, *Il-1b*, and *Tnf* mRNA in PBMCs from patients with sepsis or healthy control, supplemented with or without HET0016 (5 μM) for 24 h (*n* = 6 per group). **H** Production of MDA in PBMCs from patients with sepsis supplemented with or without HET0016 (5 μM) for 24 h (*n* = 6 per group). Data are shown as mean ± SD, and analysis by one-way ANOVA test. Data in (G, H) are analyzed by paired Student’s *t*-test. PBMCs peripheral blood mononuclear cells, MODS multiple organ dysfunction syndromes, MDA malondialdehyde, FTH ferritin heavy chain, GPX4 glutathione peroxidase 4, 4-HNE 4-hydroxynonenal. *P < 0.05, **P < 0.005, ***P < 0.0005, ****P < 0.0001.

To validate the involvement of STING-mediated ferroptosis in lethal sepsis pathogenesis, we evaluated samples from 28 patients with sepsis from our previous study [5] and 6 healthy subjects. We found that plasma levels of MDA, were strongly positively correlated with disease severity (D-lactate, APACHE II score) (Fig. 6A, B), the levels of inflammation (e.g., TNFα, IL-6), and intestinal barrier injury biomarkers (e.g., AGI score, D-lactate, intestinal fatty acid-binding protein [I-FABP]) (Supplementary Fig. S7A–D). Moreover, ferritin and MDA in plasm from patients had a reasonable performance for prediction of the 90-day mortality rate (AUC 0.819, 0.847, respectively, Supplementary Fig. S7C), and survival analysis showed significantly higher mortality with higher levels of ferritin and MDA (Fig. 6C). For a more intuitive evaluation of the intestinal injury, pathological sections from intestinal biopsy tissue of patients revealed decreased expression of FTH and GPX4 than in normal control, especially in immune cells of lamina propria; the higher level of 4-hydroxynonenal (4-HNE), an indicator of lipid peroxidation, was observed in lesion tissue than in normal tissue (Fig. 6D). Consistently, the levels of other ferroptosis-related proteins, namely, acyl-CoA synthetase long-chain family member 4, cyclooxygenase II, and 5-lipoxygenase, were correspondingly increased in patients with sepsis (Supplementary Fig. S7E). These findings suggest that ferroptosis is also involved in patients with sepsis.

Then, we assessed if ferroptosis inhibitors would work as a therapeutic strategy. Among the known inhibitors of ferroptosis, DFO is an FDA-approved iron chelator used to alleviate the iron burden in thalassemia major and sickle cell patients [30]. Unfortunately, although DFO has excellent potential as a mitigator of iron accumulation in clinical settings, its widespread use is limited by its adverse effects on the cardiovascular, respiratory, gastrointestinal, cutaneous, and nervous systems [30]. Thus, considering the possibility of clinical transformation, which depends on solubility, accessibility, targets, and research attention, we chose three different targets of compounds based on previous reports [31–33] and our above results from screened compounds: JSH-23 (a specific NF-*κ*B p65 pathway inhibitor), HET0016 (a selective 20-HETE synthase inhibitor), and carnosol (an Nrf2 activator), for further verification. Our results showed that although these three compounds remit the organ injury (Supplementary Fig. S7F), only the HET0016 improved the survival of CLP mice compared with the PBS injection group (Fig. 6E). Thus, we used HET0016 to treat the PBMC from patients with sepsis. The results showed that HET0016 could decrease the downstream activation of STING (Fig. 6F), the production of inflammatory cytokines (Fig. 6G), and MDA (Fig. 6H). Moreover, HET0016 administration decreased the level of 4-HNE, and restored the level of GPX4 and FTH in the intestine of CLP mice (Supplementary Fig. S7G). Together, these results indicated that HET0016 is a promising therapeutic regimen for improving sepsis outcomes by alleviating STING pathways activation and suppressing consequent ferroptosis.

## Discussion

**Fig. 7:**
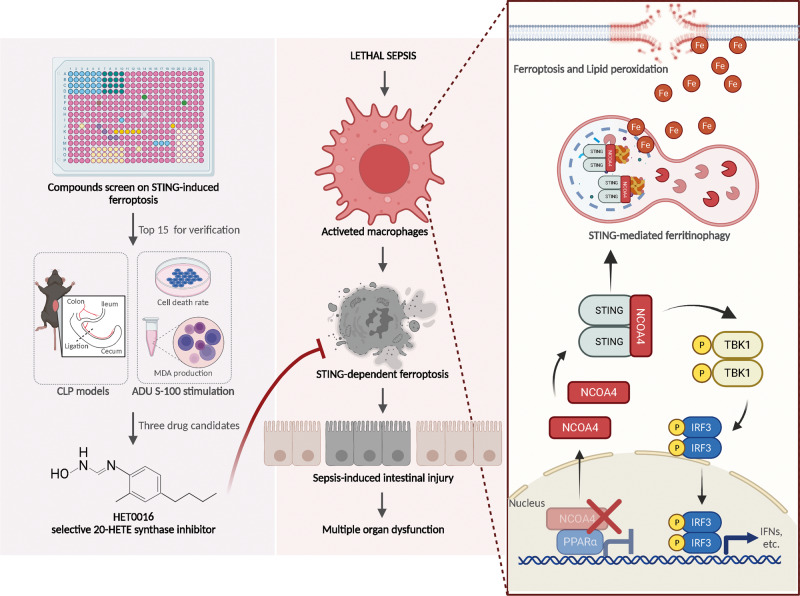
The hypothetical model for STING-regulated macrophage ferroptosis promotes sepsis-induced intestinal injury via its interaction with NCOA4. In sepsis, STING can trigger ferroptosis through direct interaction with NCOA4 to induce ferritinophagy. Mechanistically, the CBD of STING directly binds the CC domain of NCOA4, which further enhances the stability of the STING dimer and lessens the protective effects of NCOA4 as a nuclear coactivator, decreasing the nuclear localization of NCOA4. These lead to macrophage ferroptosis and further promote sepsis-induced intestinal injury. In addition, we identified potential compounds targeting STING-induced ferroptosis, which could mitigate the induction of ferroptosis and STING activation in patients and septic mice. Created with BioRender.com.

Previous studies reported that STING regulated various programmed cell death and played a vital role in health and diseases [5, 34]. However, the underlying mechanism of STING-regulated ferroptosis during sepsis has rarely been elucidated. Herein, we first reported that STING could trigger ferroptosis by directly increasing the level of intracellular iron, which is dependent on NCOA4-mediated ferritin degradation, leading to sepsis-related organ damage. Mechanistically, the CBD of STING direct binds the CC domain of NCOA4 for autophagic degradation; meanwhile, the interaction can also enhance the stability of the STING dimer and decreases the nuclear localization of NCOA4. As a result, it dampens the function of NCOA4 as a coregulator of PPARα and boosts STING-related inflammatory cascade. In addition, we identified potential compounds that could mitigate the induction of ferroptosis and STING activation in patients and septic mice (Fig. 7). These findings not only shed light on the mechanism of STING-dependent iron metabolism but also reveal ferroptosis being a potential therapeutic target for sepsis.

In past decades, STING was found to play vital roles in infection, inflammation, and immunity-related conditions. It was initially identified as a DNA sensor that controls viral infection via inducing IFN response [35], which requires cGAS to synthesize the 2’3’-cGAMP, recognized as an endogenous massager for STING signaling. Besides the cGAS-dependent pathway, bacteria-derived CDNs were also directly recognized by STING and classified as ligand-dependent activation in a cGAS-independent manner [36]. In addition, two studies uncovered the cGAS-independent activation of STING signaling by sterol regulatory element-binding protein 2–mediated trafficking [37] or ATM-IFI16 caused K63-linked ubiquitin chains on STING [38]. Moreover, recent studies reported several brand-new mechanisms of ligand-independent STING activation, including spontaneous activation of mutations in *STING1* by spontaneous rotation of the CBD [39, 40] or promoting STING polymerization and *COPI complex* (*COPA*) mutations-mediated abnormal trafficking of STING to the Golgi [41]. Likewise, several investigations also reported the IFN-independent activities and TBK1-independent phosphorylation of STING1 [42, 43]. Interestingly, we discovered that STING-mediated macrophage ferroptosis in sepsis does not rely on the classical elements of the STING pathway, including cGAS, TBK1, and cytokines transcription from NF-κB. Collectively, these findings hint that non-canonical activation and IFN-independent function of STING is also widely involved in various pathophysiological processes, while still need further studies to explore.

Recently, emerging evidence uncovered the fundamental roles of STING in regulating cell death, such as apoptosis, necroptosis, pyroptosis, ferroptosis, etc., which was also involved in physiological or pathological processes. Gulen et al. report that stimulation of STING with a lower dose stimulus triggers apoptosis in primary and malignant T cells but not in bone marrow-derived DCs or BMDMs [44]. Similarly, Wu et al. reveal that STING activities in T cells are predominantly IFN-independent, and IFN-independent activities of STING partially mediated malignant T cells substantial cell death [42]. These indicated that STING-related cell death demonstrates cellular heterogeneity in different levels of activation and disease conditions. Dr Tang’s group recently discovered that DNA damage (mitochondrial DNA and nuclear DNA) induced by cathepsin B nuclear translocation or zalcitabine/erastin-induced mitochondrial stress activates cGAS-STING to trigger autophagy-dependent ferroptosis of pancreatic cancer cells [45–47]. Our unexpected findings reveal that the direct interaction between the CC domain of NCOA4 and the CBD domain of STING but not the cGAS or TBK1 activation is the hub for STING-mediated macrophage ferroptosis in septic death. These are consistent with previous studies showing that STING activation regulates lipid peroxidation [16] and interferes with lysosomal acidification without affecting autophagosome biogenesis or fusion [48]. Meanwhile, lipid peroxidation and impaired lysosomal acidification are also the results of overloaded cytosolic free iron [12, 49]. Notably, both the CC domain of NCOA4 and the CBD domain of STING are evolutionarily conserved domains [29, 50]. Thus, CBD of STING is not only required for autophagy [50] but also essential for regulating iron metabolism. These imply that their interaction may have crucial biological functions in many physiological and pathophysiological processes related to immune metabolism.

In addition, our present study demonstrates that NCOA4 binding renders the STING dimer more stable, leading to increased levels of inflammatory cytokines, and decreased levels of nuclear NCOA4. As a result, the function of NCOA4 as a coactivator of TFs (such as PPAR) was impaired in response to STING-induced ferritinophagy. Consistently, it has been extensively reported that the expression of PPAR and its target genes are decreased in patients with sepsis, and play a protective role in the systematic inflammatory response and tissue damage [51, 52]. These findings indicated that NCOA4 is not just an adapter protein for ferritinophagy but also a regulator of immune metabolism signaling pathways.

Considering that NCOA4 plays a pivotal role in maintaining the classical function of STING and the balance of intracellular iron, which also acts as a vital trace element for the normal biological functions of cells. Therefore, a direct intervention targeting intracellular iron, NCOA4, or STING will impact normal cellular functions, such as the response to infection, the maintenance of iron metabolic balance, and enzyme activity. This is also why the therapeutic window of DFO is so narrow, as high doses significantly increase the incidence of serious adverse events or even death [53]. For these reasons, we focused on screening compounds that have robust effects in improving and protecting the antioxidant system to defend against the vicious cycle of STING-induced lipid peroxidation. Our results showed that HET0016, a selective 20-HETE synthase inhibitor, has an excellent protective performance in counteracting sepsis via inhibition of lipid peroxidation both in vitro and in vivo. Similarly, several studies also reported that HET0016 is a promising ferroptosis inhibitor for intracerebral hemorrhage treatment through decreasing the production of 20-HETE, an accelerator of lipid peroxidation, and increasing GPX4 expression [32, 54, 55]. However, further preclinical studies are needed to expatiate the pharmacokinetics, pharmacokinetics, and adverse reaction data of HET0016. In addition, some other candidate compounds related to the glucocorticoid biosynthetic process, synthesis of cholesterol, steroids, and other lipids and energy metabolism were included, although their efficiency was not significant in vivo. It indicated that metabolic pathways are closely involved in non-classical STING signaling, which still needs more investigation to elaborate.

Although we demonstrated that STING directly induces ferroptosis in a manner dependent on its interaction with NCOA4 but independent of cGAS or TBK1 in lethal sepsis, whether the role of STING upon iron metabolism orchestrates immune homeostasis still requires further investigation. Moreover, it is also unclear that there is a mechanism of inhibition of their interaction in similar conditions, such as tumor immune tolerance, immune suppression of chronic critical illness, and immune escape of virus.

In summary, we propose a novel paradigm that has never been previously reported, in which the interaction between STING and NCOA4 is central to the pathogenesis of sepsis and highlights the importance of targeting non-IFN-mediated pathway injury regulated by STING for the treatment of sepsis. Our findings also provide insights into the mechanism and potential treatment of immunopathy, such as systemic inflammatory disorders, tumor immunity, and autoimmune diseases.

## Methods

### Ethics statement

All clinical investigations were conducted according to the principles expressed in the Declaration of Helsinki. The collection of human blood samples was approved by the Institutional Review Board Ethics Committee at Jinling Hospital (2018NZGKJ-022). All patients or their legal surrogates provided written informed consent. All animal experiments were carried out in compliance with ethical regulations and were approved by the Institutional Animal Care and Use Committee of Nanjing Medical University (IACUC-2012028).

### Mice

All mice were maintained in a specific pathogen-free facility. C57BL/6 WT, *Sting1*^*–/–*^, and *Cgas*^*−/−*^ mice were purchased from the GemPharmatech Co., Ltd. *Sting1*-floxed mice were generated using a CRISPR/Cas9-based strategy by the transgenic facility at Nanjing Medical University. The fifth exon of *Sting1* was flanked by two loxP sites in *Sting1*^*f/f*^ mice. The following gRNAs were used: TCTAATCTCTCTCATCGTAC and GCTCGGGGACGCGATAGTGA. *LysM-Cre* and *Villin-cre* mice were kindly provided by Xiaoming Wang (Nanjing Medical University, Nanjing, China). *Sting1*-floxed mice were crossed with *LysM-Cre* or *Villin-cre* mice to generate macrophage-conditional *Sting1*-knockout mice (*Sting1* ^*LysM−/−*^) and intestine-conditional *Sting1*-knockout mice (*Sting1* ^*Vil−/−*^). Mice were housed with their littermates in groups of four or five animals per cage and kept on a regular 12-h light–dark cycle. All procedures for animal experiments were approved by the Ethical Review Committee for Laboratory Animal Welfare of Nanjing Medical University.

### Isolation of BMDMs

BMDMs were isolated from mice and cultured as previously described [48]. Briefly, the interior of femur and tibia were washed by placing a needle into the bone. The obtained cell suspension was lysed with RBC lysis solution (C3702, Beyotime) and then filtered with a cell strainer. Cells were resuspended in fresh DMEM supplemented with 10 ng/mL CSF-1 (315–03, PeproTech), plated in Petri dishes, and cultured at 37 °C and 5% CO_2_. After 7 days of culture, the BMDMs were mature and ready for later experimentation.

### Cell culture

RAW264.7, HEK293T cells, RAW-Lucia™ ISG cells (rawl-isg, Invivogen); RAW-Lucia™ ISG-KO-TBK1 cells (rawl-kotbk, Invivogen) and BMDMs were cultured in DMEM (G4512, Servicebio) with 10% (vol/vol) FBS (10099141, Gibco) and 100 units/mL penicillin/streptomycin (15140122, Gibco). HeLa cells were cultured in MEM (G4551, Servicebio) with 10% FBS and 100 units/mL penicillin/streptomycin. Cell lines were verified to be free of mycoplasma contamination, and the identities were authenticated by STR profiling. All cells were cultivated in a 37 °C incubator with 5% CO_2_.

Regarding the in vitro culture of PBMCs, cells were cultured in complete RPMI 1640 medium (G4532, Servicebio) with 100 units/mL penicillin, 100 μg/mL streptomycin, and 20% serum from six randomly selected patients with sepsis or age- and sex-matched healthy control (HCs) and incubated at 37 °C. For experiments involving HET0016 treatment, 5 μM HET0016 was added to PBMCs, and PBMCs were cultured for 24 h before further analysis.

### Antibodies and reagents

The antibodies used were anti-STING (19851-1-AP, Proteintech), anti-p-STING (phosphorylated at Ser365, 72971, Cell Signaling Technology), anti-p-STING (phosphorylated at Ser366, 19781, Cell Signaling Technology), anti-p-TBK1 (phosphorylated at Ser172, 5483, Cell Signaling Technology), anti-TBK1 (38066, Cell Signaling Technology), anti-p-IRF3 (phosphorylated at Ser396, 29047, Cell Signaling Technology), anti-IRF3 (4302, Cell Signaling Technology), anti-LC3A/B (12741, Cell Signaling Technology), anti-β-actin (AC026, ABclonal), anti-NCOA4 (A5695, ABclonal), anti-p62 (A19700, ABclonal), anti-DDDDK-Tag (AE063, AE005, ABclonal), anti-HA-Tag (AE008, AE036, ABclonal), anti-Myc-Tag (AE070, AE010, ABclonal), anti-GAPDH (A19056, ABclonal), anti-Lamin B1 (A16909, ABclonal), anti-FACL4 (ab155282, Abcam), anti-4 hydroxynonenal (ab46545, Abcam), anti-COX2 (ab179800, Abcam), anti-NCOA4 (ab86707, Abcam), anti-5-lipoxygenase (ab169755, Abcam), anti-FTH (ab65080, Abcam), anti-GPX4 (ab231174, Abcam), Anti-FPN1 polyclonal antibody (26601-1-AP, Proteintech), anti-ZO1 (ab221547, Abcam), anti-Occludin (ab216327, Abcam), Anti-PPAR alpha (ab227074, Abcam), anti-p-p65 (phosphorylated at Ser536, ab76302, Abcam), anti-p65 (ab32536, Abcam), Alexa Fluor® 647 anti-mouse IgG (ab150115, Abcam), and Alexa Fluor® 555 anti-rabbit IgG (ab150074, Abcam), anti-IL-6 (GB11117, Servicebio), and anti-IL-1 beta (GB11113, Servicebio). Antibodies were diluted according to the manufacturer’s instructions.

The following were used: DMXAA (HY-10964, MedChemExpress), di-ABZI (HY-112921B, MedChemExpress), c-di-AMP sodium (CDA, HY-12326A, MedChemExpress), ADU-S100 disodium salt (HY-12885A, MedChemExpress), CQ (HY-17589A, MedChemExpress), Ferrostatin-1 (HY-100579, MedChemExpress), Liproxstatin-1 (HY-12726, MedChemExpress), RSL3 (HY-100218A, MedChemExpress), DFO (HY-B0988, MedChemExpress), C-176 (HY-112906, MedChemExpress), Bafilomycin A1 (HY-100558, MedChemExpress), HET0016 (HY-124527, MedChemExpress), Carnosol (HY-N0643, MedChemExpress), JSH-23 (HY-13982, MedChemExpress), and Lipofectamine™ 3000 transfection reagent (L3000150, Invitrogen).

### The CLP mouse model

Sepsis was induced through a surgical procedure termed CLP as described previously [48]. Briefly, the mouse was anesthetized, and a small midline abdominal incision was made. The cecum was then exteriorized, and the distal three-quarters of the cecum was immediately ligated with 3-0 silk without causing intestinal obstruction. The ligated cecum was punctured with an 18-gauge needle, and a small amount of feces was gently squeezed out of the perforation to ensure patency of the punctures. The cecum was then relocated into the abdominal cavity, and the incision was closed. In the sham surgical controls, the cecum was exposed without ligation or puncture. All mice received 0.3 mL PBS after surgery for fluid resuscitation.

At indicated time points, mice were euthanized and intestine, liver, lung, and peripheral blood were collected for analysis. Plasma was obtained from anticoagulated whole blood after removal of the blood cells by centrifugation (3000 × g, 10 min) at 4 °C and then used for the measurement of cytokines. Intestine tissue was cut into pieces and frozen in liquid nitrogen before storage at –80 °C. Intestine, liver, and lung tissue were fixed in 4% PFA solution and then for histological staining. Survival analysis was observed for up to 14 days. Mice were treated with C-176 (13.42 mg/kg), LIP-1 (10 mg/kg), RSL3 (10 mg/kg), DFO (100 mg/kg), HET0016 (10 mg/kg), JSH-23 (20 mg/kg), or Carnosol (10 mg/kg) at 2 h before CLP and 12, 24, 48, and 72 h after CLP.

### Tissue dissociation and single-cell suspension preparation

The intestine of the control and CLP-treated group from WT and Sting1^*−/−*^ mice were removed at the indicated time (*n* = 3 for each group). The fresh tissues were washed with Hank’s balanced salt solution (HBSS) three times and then minced into 1–2 mm pieces. The tissue pieces were digested with 2 mL CelLiveTM Tissue Dissociation Solution (Singleron) by Singleron PythoN™ Automated Tissue Dissociation System (Singleron) at 37 °C for 15 min. Afterward, cells were incubated with red blood cell lysis buffer for another 10 min. The solution was then centrifuged at 500 g for 5 min and suspended softly with PBS. Finally, the samples were stained with trypan blue (Sigma, United States), and the cellular viability was evaluated microscopically.

### Library preparation and scRNA-seq

Single-cell suspensions (1 × 10^5^ cells/mL) with PBS were loaded into a microfluidic chip using the Singleron Matrix^®^ Single Cell Processing System (Singleron). Subsequently, the scRNA-seq libraries were constructed according to the protocol of the GEXSCOPE^®^ Single Cell RNA Library Kits (Singleron) [56]. scRNA-seq libraries were diluted to 4 nM and pooled for sequencing. At last, pools were sequenced on Illumina HiSeq X with 150 bp paired-end reads.

### scRNA-seq quantifications and statistical analysis

Seurat (version 4.0.5) [57] was used for single-cell data processing. The raw data for each sample generated by single-cell sequencing was imported into Seurat with at least 300 genes present in each cell and at least 10 cells. For each cell, the number of genes and UMIs were quantified, and cells with the detection threshold of 500–30,000 UMIs were retained. Cells with high detection rates of mitochondrial gene expression were excluded.

To perform integration of different datasets, each raw expression matrix was logged normalized, and 3000 highly variable genes were identified for each dataset. Subsequently, anchors between datasets were identified followed by data integration. SCTransform [58] normalization was applied during the integration to remove the influence of technical characteristics.

Dimension reduction was performed on the integrated datasets for further cluster analysis. Principal component analysis (PCA) was performed using the 3000 most variable genes. JackStraw function was used to determine the statistical significance of PCA scores. The most significant principal components (PCs) were used as input for Uniform Manifold Approximation and Projection to visualize the data in a two-dimensional space. For clustering, the FindClusters function based on shared nearest neighbor modularity optimization was applied on the most significant PCs. Clusters in the integrated datasets were annotated by their expression profiles of genes in the known maker lists.

Differential gene expression analysis between single-cell groups was performed using the FindMarkers function with the default configurations. Down sampling was applied during the differential analysis. GSVA implemented in the GSVA package [59] was used for GSEA, and *p* values were calculated with the moderated *t*-tests in the limma R package. Adjusted *p* values were calculated by the false discovery rate (Benjamini–Hochberg) correction method. KEGG, REACTOME, BIOCARTA, and WP subcollections of C2 curated gene sets from MSigDB [60, 61] database and ferroptosis-related genes curated by us were used for GSVA. Ferroptosis-related genes were obtained from GENE DATABASE (https://www.genecards.org/) [62] by searching the keywords “Ferroptosis” and other related literature. Consequently, the 170 ferroptosis-related genes were included in the analysis and are provided in Supplementary Table S1.

### RNA isolation and real-time quantitative PCR

Circulating DNA was isolated from 200 μL of plasma using the QIAamp DNA Blood Mini Kit (Qiagen) according to the manufacturer’s instructions. RNA was extracted from cells or tissue. Briefly, 1 mL of TRIzol containing the sample was mixed with 200 μL of trichloromethane. Samples were centrifuged at 12,000 × g for 15 min at 4 °C. The upper aqueous phase was mixed with 500 μL of isopropanol and precipitated for 10 min. Precipitated RNA was pelleted at 12,000 × g for 10 min, washed once with 70% ethanol (7500 × g for 5 min), and resuspended in RNase-free H_2_O. RNA was then reverse-transcribed into DNA using HiScript III RT SuperMix (R323, Vazyme). Mitochondrial genes (*D-loop* and *ND*_*2*_) were used to quantify cell-free mtDNA. The reverse transcription products of each sample were amplified by QuantStudio™ 3 (Applied Biosystems) using SYBR Green QPCR Master Mix (Q711, Vazyme) according to the manufacturer’s instructions. All the samples were run in triplicate. The data were normalized to *β-actin*, and the fold change was calculated via the 2^−△△Ct^ method. Relative mRNA concentrations are expressed in arbitrary units compared to those in the untreated group, which was assigned a value of 1.

### Enzyme-linked immunosorbent assay

Commercially available ELISA kits were used to measure the concentrations of IL-6 (CSB-E04639m, CUSABIO), TNF-α (CSB-E04740h or CSB-E04744m, CUSABIO), I-FABP (CSB-E08024h), D-lactate (HM11235, Bio-Swamp), and ferritin (ab108837, ABCAM) in the indicated samples according to each manufacturer’s instructions.

### Immunoprecipitation analysis

Cells were lysed at 4 °C in ice-cold lysis buffer (P0013J, Beyotime) with protease inhibitor cocktail (HY-K0010, MedChemExpress), and cell lysates were cleared by a brief centrifugation step (12,000 × g, 10 min). Prior to immunoprecipitation, samples were precleared with protein A/G agarose beads (SA032005, SMART Lifesciences) at 4 °C for 0.5 h. After removing these beads from the lysates, we incubated the lysates with specific antibodies overnight at 4 °C with gentle shaking. Protein A/G agarose beads were added to the mixtures for 2 h at 4 °C. After three washes with ice-cold lysis buffer, the proteins were eluted from the beads into the loading buffer (P0015B, Beyotime), and the samples were boiled for 10 min at 100 °C. The proteins were then analyzed by western blotting.

### Western blotting

Tissue or cells were lysed in RIPA lysis buffer (P0013B, Beyotime) with protease inhibitor cocktail (HY-K0010, MedChemExpress), PMSF (ST506, Beyotime), and phosphatase inhibitor cocktail (HY-K0021, MedChemExpress) for 20 min at 4 °C. The lysates were centrifuged at 12,000 g at 4 °C for 15 min, and the supernatants were mixed with 5X sodium dodecyl sulfate (SDS) loading buffer (containing β-mercaptoethanol) and boiled for 10 min at 100 °C. For the STING dimerization assay, supernatants were mixed with a loading buffer that lacked β-mercaptoethanol.

Proteins were resolved by SDS-polyacrylamide gel electrophoresis (PAGE) and transferred onto a polyvinylidene fluoride membrane (Millipore). After blocking with Tris-buffered saline Tween 20 (TBST) containing 5% nonfat dry milk for 1 h at room temperature, the membrane was incubated with the indicated primary antibody overnight at 4 °C. Then, the membrane was washed with TBST and incubated with a secondary antibody (7074 or 7076, Cell Signaling Technology) for 1 h at room temperature. The blots were imaged using a Tanon 5200 chemiluminescent imaging system (TANON, Shanghai, China) and quantified with ImageJ software version 1.49 (National Institutes of Health, Bethesda, MD, USA). Full and uncropped western blots used in this article are uploaded as Supplementary material.

### Histological analysis

The tissue was fixed in 4% buffered formaldehyde for histology and embedded in paraffin. Tissue sections were stained with H&E. To evaluate the intestinal injury, Chui’s scoring system was used as previously described [15]. To evaluate lung inflammation and damage, the entire lung surface was analyzed with respect to the following parameters: neutrophils in the alveolar space, neutrophils in the interstitial space, hyaline membranes, proteinaceous debris filling the airspaces, and alveolar septal thickening. Each parameter received a score from 0 (absent) to 2 (severe). Liver inflammation was scored according to the following parameters: number of thrombi, number of (micro)abscesses, presence and degree of inflammation, and presence and degree of necrosis. Each parameter received a score from 0 (absent) to 3 (severe). Images were acquired with a Nikon 50i inverted microscope.

### Immunohistochemistry (IHC) and immunofluorescent (IF) analyses

IHC and IF staining were performed as previously described [48]. Briefly, tissue samples were fixed in 4% paraformaldehyde and embedded in paraffin. Tissue sections on slides were incubated at 4 °C overnight with the indicated primary antibody and then incubated with the relevant secondary antibody. For IHC staining, antigens were visualized using the hydrogen peroxidase substrate 3,3’-diaminobenzidine, which resulted in brown labeling of immunoreactive cells. For IF staining, the nuclei were counterstained with 4’,6-diamidino-2-phenylindole (G1012, Servicebio). Slides were dried and mounted using ProLong Antifade mounting medium (G1401, Servicebio) and visualized using a Nikon fluorescence microscope or a Zeiss LSM 980 laser scanning confocal microscope. ZEN (blue edition, v3.0) was used for 2.5D display of NCOA4 localization. The peak values represent fluorescence intensity.

### Transmission electron microscopy (TEM)

Cells and tissue sections were collected and incubated overnight in 0.1 M PBS (pH 7.4) containing 2.5% glutaraldehyde, followed by fixation in 1% osmium tetroxide for 1 h. After dehydration in a graded ethanol series and embedding in araldite, the ultrathin sections (70–90 nm) were stained with uranyl acetate and lead citrate and then viewed under an H-7650C TEM (Hitachi, Japan).

### RNA interference (RNAi)

The siRNAs used in this study were synthesized by GENERAL BIOL (Chuzhou, China). Cells were transfected with RNAi oligonucleotides using Lipofectamine 3000 reagent (Invitrogen) or INTERFERin^®^ (Polyplus-transfection) according to the manufacturer’s instructions. The corresponding siRNAs used were as follows:

m-siNCOA4-1, forward: 5’-ACAAGAAAUUGCUGGAAAATT-3’;

m-siNCOA4-2, forward: 5’-GUGGAUGGCAGCUGGGAAATT-3’;

m-siNCOA4-3, forward: 5’-GGAAAGGACAAGAAUGGAATT-3’,

h-siNCOA4-1, forward: 5’-GGAAGTGCCTGGTACTGAA-3’;

h-siNCOA4-2, forward: 5’-CAACTGTCCTGCTCTTGA-3’;

h-siNCOA4-3, forward: 5’-GGCAATGACTCCTTCTAGA-3’.

### Plasmids and transfection

The *STING1*-Flag plasmid was obtained from Sino Biological (HG29810-CF). The following STING1 fragments were subcloned into the pCMV3-C-Flag vector, with the Flag tag oriented at the C-terminus: those containing certain domain deletions, namely, ΔTM (amino acids 147–379), ΔDD + CBD (amino acids 1–146 + 341–379), and ΔCTT (amino acids 1–340); the fragment containing only the CBD (amino acids 180–340); and those containing mutants C88S/C91S and S366A. The DNA for the full-length human NCOA4 isoform (amino acids 1–614, NM_001145263.1) and fragments of the NCOA4 corresponding to amino acids 1–245, 246–425, and 426–614 were cloned into the pCMV3-C-Myc vector using Gateway recombination. Constructs were transfected into the indicated cell lines by transient transfection using Lipofectamine 3000.

### Cell death analysis

BMDMs and RAW264.7 cell death were assessed using the LDH assay (G1780, Promega) according to the manufacturer’s protocol. Following experimental treatment, 50 μL of the supernatant of each sample was transferred to a 96-well plate, 50 μL of CytoTox 96^®^ reagent was added to each well, and the plate was incubated for 30 min at room temperature. Next, a stop solution was added, and the absorbance at 490 nm was measured by a plate reader (SpectraMax iD5, Molecular Devices). The results were calculated according to the following formula: cell death ratio % = (absorbance of processed sample − absorbance of sample control well)/(absorbance of cell maximum enzyme activity − absorbance of sample control well) × 100%.

BMDMs and RAW264.7 cell death were also measured by using propidium iodide (PI) cell death assay kit (G1021, Servicebio) according to the manufacturer’s protocol. Briefly, cells (3 × 10^5^/well) were seeded in 12-well cell culture plates and incubated in a humidified atmosphere with 5% CO_2_ at 37 °C. After 24 h, cells were treated with DMXAA (75 µg/mL), c-di-AMP, or di-ABZI (10 µM) in the absence or presence of Fer-1 (10 µM) or DFO (100 nM) for 16 h. Then, cells were rinsed with warm PBS, and cell viability was assessed by PI staining. Morphological changes were examined with an EVOS™ M5000 fluorescence microscope.

### Labile iron imaging

A total of 3 × 10^5^ RAW264.7 cells were seeded on glass-bottom culture dishes (801002, NEST). After treatment with the indicated drugs, the cells were washed with HBSS three times. Next, the cells were stained with 1 μM FerroOrange (F374, Dojindo) in HBSS for exactly 30 min at 37 °C and immediately imaged. Focal images were acquired with a Zeiss LSM 980 inverted laser scanning confocal microscope (Zeiss).

### Lipid peroxidation assessment

The relative MDA concentrations in cell lysates were assessed using a Lipid Peroxidation MDA Assay Kit (S0131S, Beyotime) according to the manufacturer’s instructions. Briefly, cells were added to lysis buffer on ice, homogenized, and centrifuged at 12,000 × g for 10 min at 4 °C to collect the supernatant. One hundred microliters of the supernatant were incubated with 200 μL of the test solution for 15 min at 100 °C and then cooled to room temperature. The mixture was centrifuged at 1000 × g for 10 min to obtain the supernatant, and the absorbance at 532 nm was read using a microplate reader (SpectraMax iD3, Molecular Devices).

To visualize lipid peroxidation, cells were seeded in 12-well plates. After the corresponding treatments, cells were stained with 10 μM Liperfluo (L248; Dojindo Molecular Technology) for 30 min at 37 °C. Then, the cells were washed with HBSS, the medium was replaced, and the cells were examined using a fluorescence microscope (EVOS™ M5000, Thermo Fisher).

### Total GSH and GSH/GSSG assays

Intestinal tissues were homogenized, and the supernatants were collected for GSH analysis using a total GSH and GSH/GSSG assay kit (S0052, S0053, Beyotime) according to the analytical protocol provided by the manufacturer.

### Patients

The collection of human blood samples was approved by the Institutional Review Board Ethics Committee at Jinling Hospital (2018NZGKJ-022) and was conducted in accordance with the Helsinki Declaration (WMA Declaration of Helsinki, 2013). Before collecting blood samples, informed consent was obtained from all human participants. 28 patients involved in this study were recruited from December 2018 to March 2019, all fulfilling the diagnosis of sepsis according to the third international consensus definitions for sepsis and septic shock (Sepsis-3) [2]. Blood samples were collected 1 day after admission to the surgical intensive care unit. Then, serum was obtained by centrifuging blood samples at 3000 × g at 4 °C for 10 min and stored at −80 °C until further analysis. HCs were age- and sex-matched individuals without autoimmune, inflammatory, or infectious diseases. Clinical characteristics are presented in Supplementary Table S2.

### PBMCs isolation

Human PBMCs were isolated from the blood of sepsis patients and healthy donors. The use of PBMCs complied with institutional ethics guidelines and approved protocols of Jinling Hospital. PBMCs were collected from patients with sepsis and HCs by density gradient centrifugation. Briefly, 4 mL of Lymphoprep^TM^ (07801, STEMCELL) was added to a 15-mL SepMate^TM^ tube (85415, STEMCELL). Two milliliters of blood were diluted with 2 mL of PBS, and the blood was layered on top of Lymphoprep^TM^. Next, the mixture was centrifuged at 800 × g for 20 min at room temperature with the brake off. The upper plasma and PBMC layers were removed by centrifugation at 800 × g for 3 min at room temperature for subsequent analyses.

### Mass spectroscopy

PBMCs were washed with PBS and lysed for immunoprecipitation using anti-STING antibody. Immunoprecipitates were separated by SDS–PAGE and then Coomassie blue staining was carried out. Then, the gel was washed twice and destained at room temperature for 30 min. After dehydration and reduction, the gel was subjected to tryptic digestion for 16 h at 37 °C. The peptide extract and the supernatant of each gel spot were combined and then completely dried.

Samples were resuspended in nano-HPLC buffer and loaded on a Trap column (100 µm × 20 mm, RP-C18, Thermo Inc.) with an autosampler and separated with an analytical column (75 µm × 150 mm, RP-C18, Thermo Inc.) at 300 nL/min with a 30-min mobile-phase gradient between samples with additional blank solvent cleaning. Enzymatic hydrolysis was performed with an LTQ Orbitrap Velos Pro (Thermo Finnigan). The experimental period was 105 min, and cation detection mode was used. Data were acquired using an ion spray voltage of 1.8 kV and an interface heater temperature of 150 °C. A standard was used for calibration before samples were run with a parent ion scanning range of 350–1800 m/z. For information-dependent acquisition, the strongest 15 pieces of the map (MS2 scan) after each full scan carried out with the following parameters were acquired: fracture mode of collision-induced dissociation, normal chemical energy of 35%, *q* value of 0.25, activation time of 30 ms, and dynamic exclusion set to ½ of the peak width (30 s). The MS resolution was 60,000 with an M/Z of 400, and the MS/MS resolution was the unit mass that was resolved in the ion trap. The precursor was then removed from the exclusion list. MS was carried out using profile model collection, and MS/MS data were collected with the centroid method to reduce file size. Proteins were successfully identified based on 95% or higher confidence intervals based on their scores in the MASCOT V2.3 search engine (Matrix Science Ltd., London, UK).

### Cytosolic and nuclear fractionation for protein isolation

Cell cytoplasmic/nuclear fraction for protein isolation was performed using a Cytoplasmic & Nuclear Protein Extraction Kit (P0027, Beyotime) according to the manufacturer’s protocol. Briefly, cells were lysed in cytosolic extraction buffer on ice for 15 min, vortexed for 5 s, and centrifuged at 12,000 rpm for 5 min. The supernatant was collected to obtain the cytosolic fraction. The pellet was then lysed in nuclear extraction buffer on ice for 30 min with vortexing every 2 min and then centrifuged at 16,000 rpm for 10 min. The supernatant was collected to obtain the nuclear fraction. Loading buffer (P0015L, Beyotime) was added to each cytoplasmic or nuclear fraction sample, and the samples were boiled for 10 min at 100 °C for SDS–PAGE and western blot analysis.

### Simulation of the interaction between STING and NCOA4

The interaction between STING (PDB ID: 6S26) and NCOA4 (AlphaFold ID: AF-Q13772-F1) was simulated using the ZDOCK module of Discovery Studio 2019. Specifically, STING was set as the ligand, and NCAO4 was used as the receptor with an angular step size of 6 for final conformational sampling. Two hundred poses were generated for each docking process and clustered using an RMSD cutoff value of 10 and an interface cutoff value of 10. The maximum number of clusters was set to 100. Poses were ranked by ZDOCK score. The top 100 predicted docked conformations were subjected to refinement and re-ranking using the RDOCK module to remove clashes and optimize polar and charge interactions to help identify a near-native conformation. The final docked conformations with the best ZDOCK and RDOCK scores among all refined conformations from each docking process were chosen.

### Bioactive compound library profiling in cells

Large-scale bioactive compound profiling was conducted as described previously and below [32]. A bioactive compound library (HY-L037, MedChemExpress) containing 625 compounds that target redox-related pathways was used, and each compound was stored in 10 mM dimethyl sulfoxide. For the primary screen, compounds were prediluted to 5 µM in high-glucose DMEM. A total of 2 × 10^4^ RAW264.7 cells were seeded per well in 96-well plates. Cells were incubated overnight at 37 °C with 5% CO_2_ before the experiments were conducted. The next day, the medium was replaced with 100 μL of medium containing the indicated compound and incubated at 37 °C and 5% CO_2_ for 1 h. Then, 25 μL of medium containing ADU-S100 (145 µM) was added (final concentration, 29 µM), and cells were incubated at 37 °C with 5% CO_2_ for 24 h. Cell death was confirmed by LDH assay (G1780, Promega). The percent cell death was calculated based on cell death in the ADU-S100-treated wells, which was set to 100%. For illustration purposes, the log2 change was calculated. The top 15 compounds that reduced or increased cell death in this analysis were evaluated again in BMDMs. Assays were performed as described for the primary screen.

### Statistical analysis

Data in the figure legends are presented as the mean ± SD. Each experiment was repeated at least three times independently, and similar results were obtained. All data were analyzed using GraphPad Prism 9 software. The Shapiro–Wilk test was used for normal distribution verification. For data with a normal distribution and homogeneity of variance, the independent sample *t*-test was used to compare the differences between the two groups. In some experiments, the paired Student’s *t*-test was applied as indicated. For data with a nonnormal distribution, the Mann–Whitney test or Kruskal–Wallis non-parametric test were applied. One-way analysis of variance was used for comparisons among multiple groups. Correlation analysis was carried out with Pearson’s correlation coefficient or Spearman correlation analysis as indicated. The Kaplan–Meier method was used to compare differences in mortality rates between groups. In general, * indicates *p* < 0.05, ** indicates *p* < 0.01; *** indicates *p* < 0.001, and **** indicates *p* < 0.0001.

## Supplementary information

Supplementary Figures S1–S7 Tables S1 and S2

Reproducibility checklist form

This file contains western blot full scans

## Data Availability

scRNA-seq data: Gene expression. Deposited at the National Center for Biotechnology Information Sequence Read Archive (https://www.ncbi.nlm.nih.gov/sra) under accession number SRR18159260 to SRR18159271.
