# Supplementary material for: The interaction between STING and NCOA4 exacerbates lethal sepsis by orchestrating ferroptosis and inflammatory responses in macrophages
Source: Cell Death Dis. 2022 Jul 28;13(7):653. doi: 10.1038/s41419-022-05115-x (PMC9334269; doi:10.1038/s41419-022-05115-x)
Supplement: Supplementary file 1 — Supplementary Figures S1–S7 Tables S1 and S2 [file 41419_2022_5115_MOESM1_ESM.pdf]

Supporting information for:

The interaction between STING and NCOA4 exacerbates lethal sepsis by orchestrating ferroptosis and inflammatory responses in macrophages

TABLE OF CONTENTS

SUPPLEMENTARY FIGURES·· 2

SUPPLEMENTARY TABLES·· 10

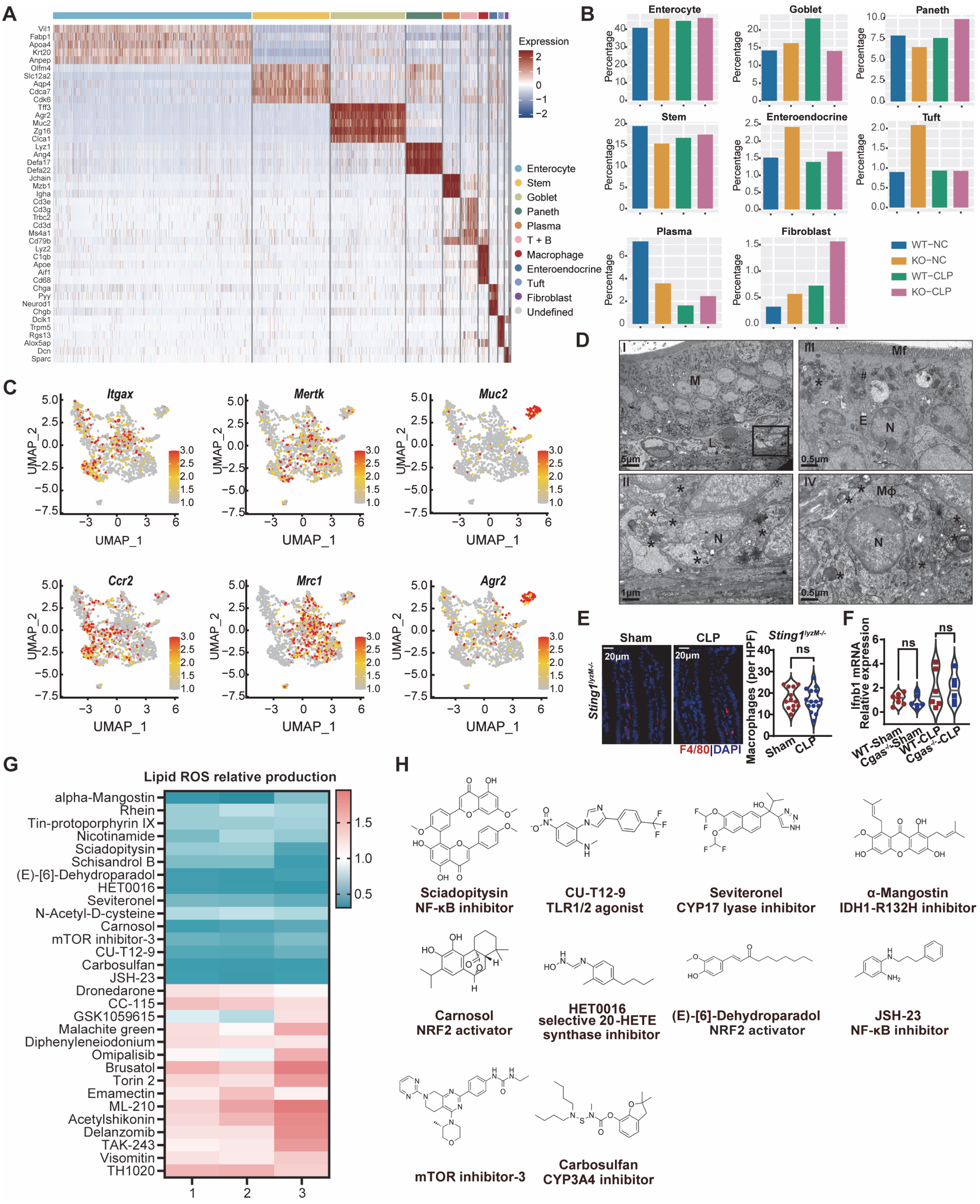

Figure S1. *Sting1* deficiency protects against septic death via mitigating lipid peroxidation. (**A**) Cell-type signatures. Heatmap shows the relative expression levels of cell-type signature genes (rows) across cells (columns) sorted by cell types from intestine of WT or *Sting1*$^{-/-}$ mice subjected with or without CLP model (n = 3 per group). (**B**) Bar plots compare the percentages of the enterocytes, fibroblasts, goblet, paneth, stem, enteroendocrine, tuft and plasma cells in WT or *Sting1*$^{-/-}$ mice subjected with or without CLP (n = 3 per group). (**C**) UMAP visualization of the

expression of M1 macrophage markers (*Itgax*, *Ccr2*), M2 macrophage markers (*Mertk*, *Mrc1*) and goblet-like macrophage markers (*Agr2*, *Muc2*) in 1341 macrophages. (**D**) Representative transmission electron microscope images of the mice showing intestinal barrier at 24 h after CLP: I, the image of the intestinal barrier, including mucosa layer and lamina propria; II, the enlarged image from I as indicated; III, a representative image of enterocytes; IV a representative image of macrophage in submucosa layer. M, mucosa layer; L, lamina propria; N, cell nucleus; E, enterocytes; Mf, microfold; Mφ, macrophage; *, injury mitochondria; #, normal mitochondria. The scale bar represents 0.5 μm. (**E**) Effects of sepsis on the counts of macrophages in small intestine of $Sting1^{LysM-/-}$ mice (n = 3 per group) at 24 h after CLP. Cells were counted in each of 15 randomly selected microscopic field (×400). (**F**) qPCR analysis of *Ifnb1* of intestinal tissue as indicated (n = 4 per group) (**G**) Heatmap of lipid ROS production fold change in RAW264.7 cells after ADU-S100 (29 μM) stimulation in the absence or presence of indicated compounds at 24 h (n = 3 per group). (**H**) Structures for selected compounds.

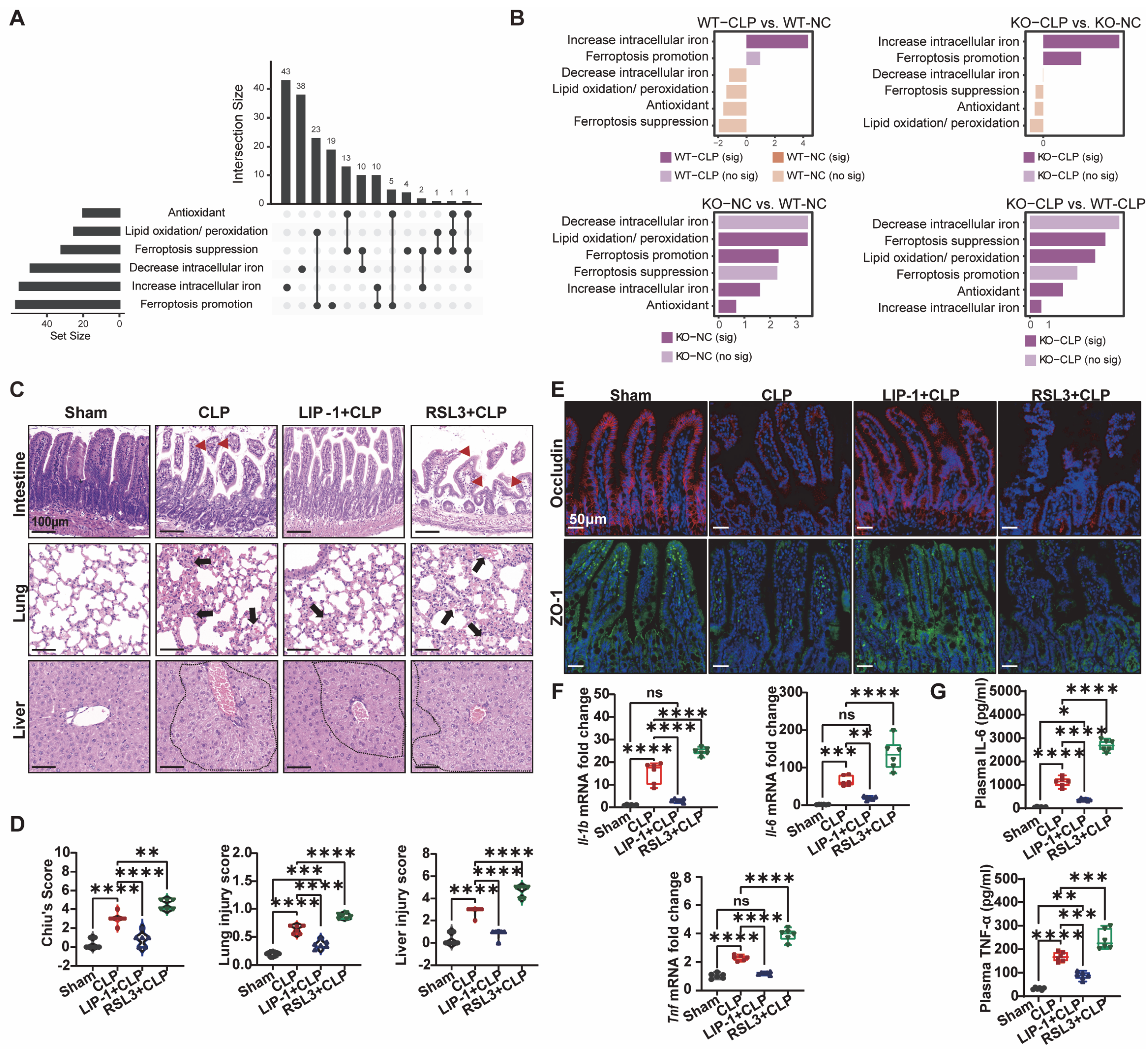

Figure S2. STING drives sepsis-induced tissue damage through triggering ferroptosis. (**A**) Upset plot illustrates unique and intersect genes of ferroptosis-related gene sets. Bar plots illustrate the numbers of genes in the corresponding subsets of unique and intersect genes. (**B**) Ferroptosis-related GSVA enrichment score under different conditions. Sig, significantly enriched; no sig, not significantly enriched. (**C-D**) Representative histopathological section images (**C**) and Histopathological scoring (**D**) of intestine, lung, and liver at 24 h after CLP (n = 6 per group). (**E**) Representative Occluding (red) and ZO-1 (green), and DAPI (blue) immunofluorescence images from indicated mice at 24 h after CLP (n = 6 per group). (**F**) qPCR analysis of *Il-1b*, *Il-6*, *Tnf* mRNA of intestinal tissue as indicated at 24 h after CLP (n = 6 per group). (**G**) ELISA for plasma level of IL-6 and TNF-α in indicated groups at 24 h after CLP (n = 6 per group). Data are shown as mean ± SD. Data are analyzed by using one-way ANOVA test (**D**, **F**, and **G**). GSVA: gene set variation analysis; CLP: cecal ligation and puncture; LIP-1: liproxstatin-1; DFO: deferoxamine; MDA: malondialdehyde; BMDMs: bone marrow-derived macrophages; ANOVA: analysis of variance. *P <0.05, ** P <0.005, *** P < 0.0005, **** P < 0.0001.

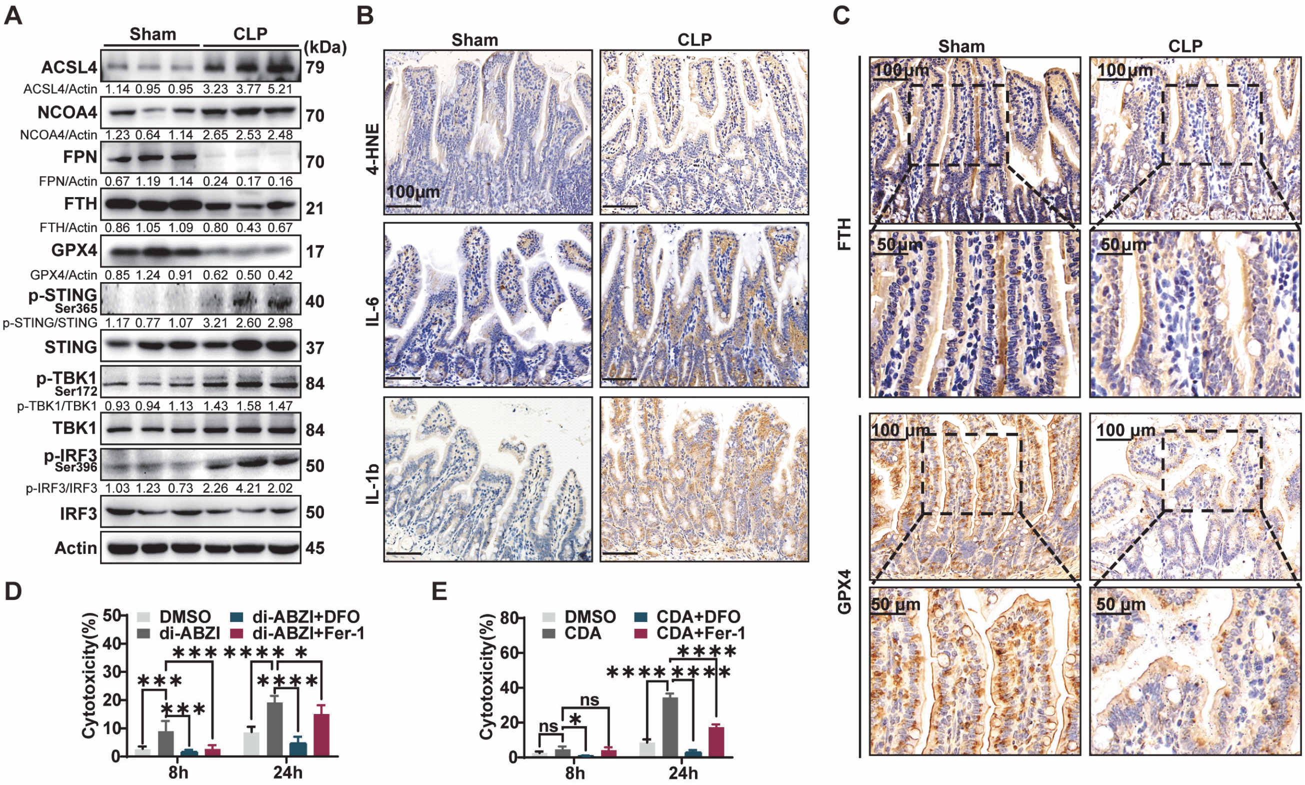

Figure S3. STING activation could induce the macrophages death, which can be rescued by ferroptosis inhibitors. (**A**) Representative immunoblot for STING and ferroptosis pathway in the intestinal tissue of WT mice at 24 h after CLP (n = 3 per group). (**B-C**) Representative immunohistochemical images of indicated protein in the intestinal tissues of WT mice at 24 h after CLP. (**D-E**) Percentage of LDH release in RAW264.7 cells stimulated with di-ABZI (10 μM) or CDA (20μM) in the absence or presence of Fer-1 (10 μM) or DFO (100 nM) at different time points. Data are shown as mean ± SD, and analysis by using one-way ANOVA test (**D** and **E**). CDA: c-di-AMP; LDH: lactate dehydrogenase; DFO: deferoxamine; ANOVA: analysis of variance. **P* <0.05, ** *P* <0.005, *** *P* < 0.0005, **** *P* < 0.0001.

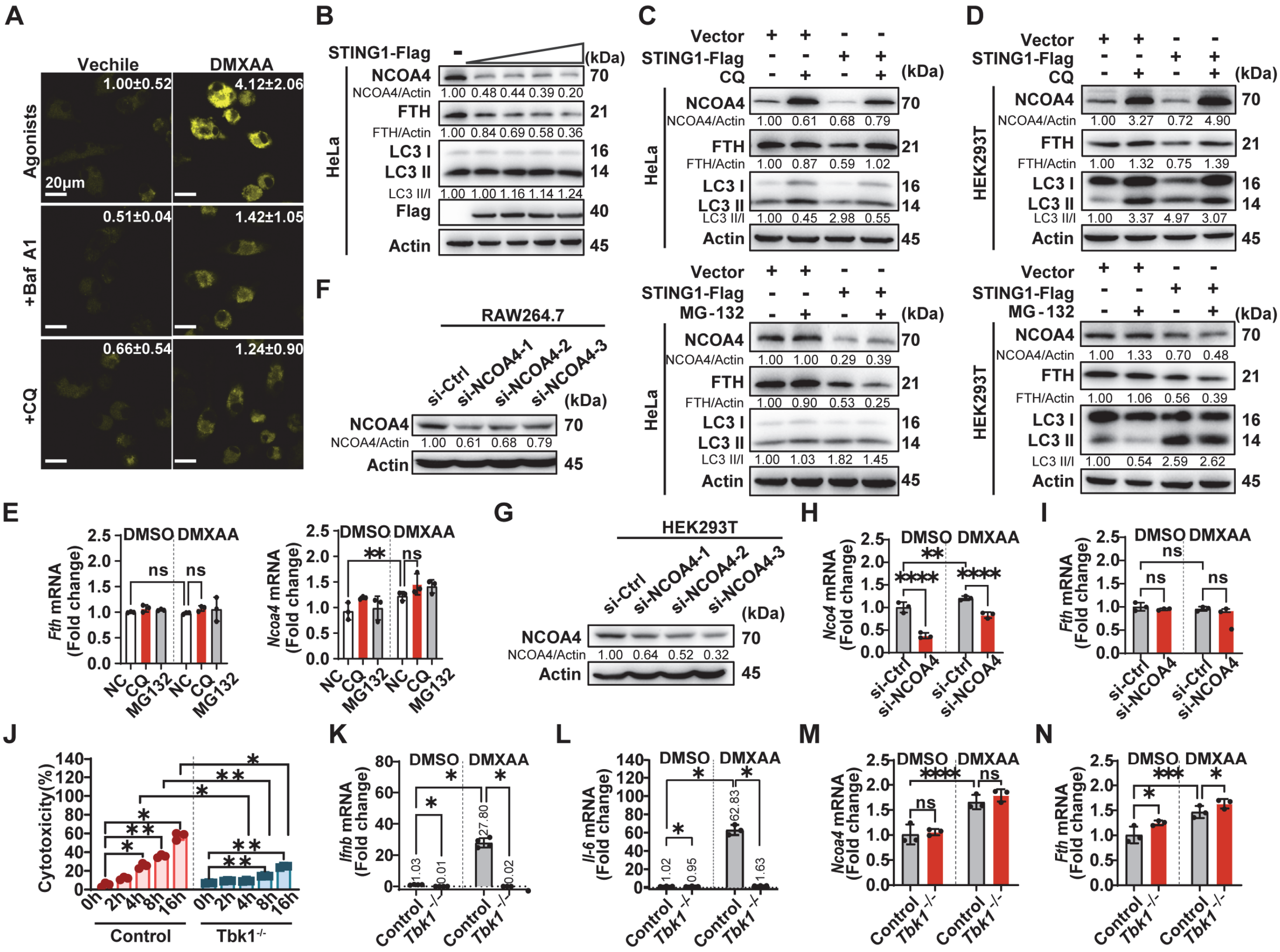

Figure S4. Ferritinophagy induced by STING is independent of TBK1. (**A**) Ferro-orange (1 μM) staining for intracellular Fe^{2+} in BMDMs treated with DMXAA (75 μg/ml) the presence or absence of BafA1 (50 nM) or CQ (10 μM) for 16 h. Typical changes in fluorescence are shown. The scale bar represents 20 μm. The fold change of integrated density in each group was indicated at the upper-right corner (**B**) Immunoblot analysis of indicated proteins in HeLa cells transfected with increasing concentration of *STING1*-Flag plasmid (0.25-1.50 μg). (**C-D**) Immunoblot analysis of indicated proteins in HeLa cells (**C**) or HET293T cells (**D**) transfected with *STING1*-Flag plasmid (1.50 μg) in the presence or absence of CQ (10 μM) or MG-132 (1 μM) for 24 h. (**E**) qPCR analysis of *Fth* and *Ncoa4* mRNA in RAW264.7 cells treated with 75 μg/ml DMXAA in the presence or absence of CQ (10 μM) or MG-132 (1 μM) for 16 h. (**F-G**) Validation of siRNA efficiency by western blotting in RAW264.7 (**F**) or HEK293T cells (**G**) at 48h after transfected with siRNA (50 nM). (**H-I**) qPCR analysis of *Ncoa4* and *Fth* mRNA in in RAW264.7 cells transfected with si-NCOA4 (50 nM) for 36 h and treated with 75 μg/ml DMXAA for 16 h. (**J**) Percentage of LDH release in $Tbk1^{-/-}$ RAW264.7 cells stimulated with DMXAA (75 μg/ml) at indicated time points (n = 3 per group). (**K-N**) qPCR analysis of *Ifnb* (**K**), *Il-6* (**L**), *Ncoa4* (**M**), and *Fth* (**N**) mRNA in the indicated treatment groups treated with 75 μg/ml DMXAA for 16 h. All are representative of at least three independent experiments with similar results. Data are shown as mean ± SD, and analysis by two-way ANOVA test (**E**, **H**, **I**, and **J-N**). LDH: lactate dehydrogenase; DFO: deferoxamine; Baf A1: bafilomycin A1; CQ: chloroquine; TBK1: TANK-binding kinase 1; ANOVA: analysis of variance. *P <0.05, ** P <0.005, *** P < 0.0005, **** P < 0.0001.

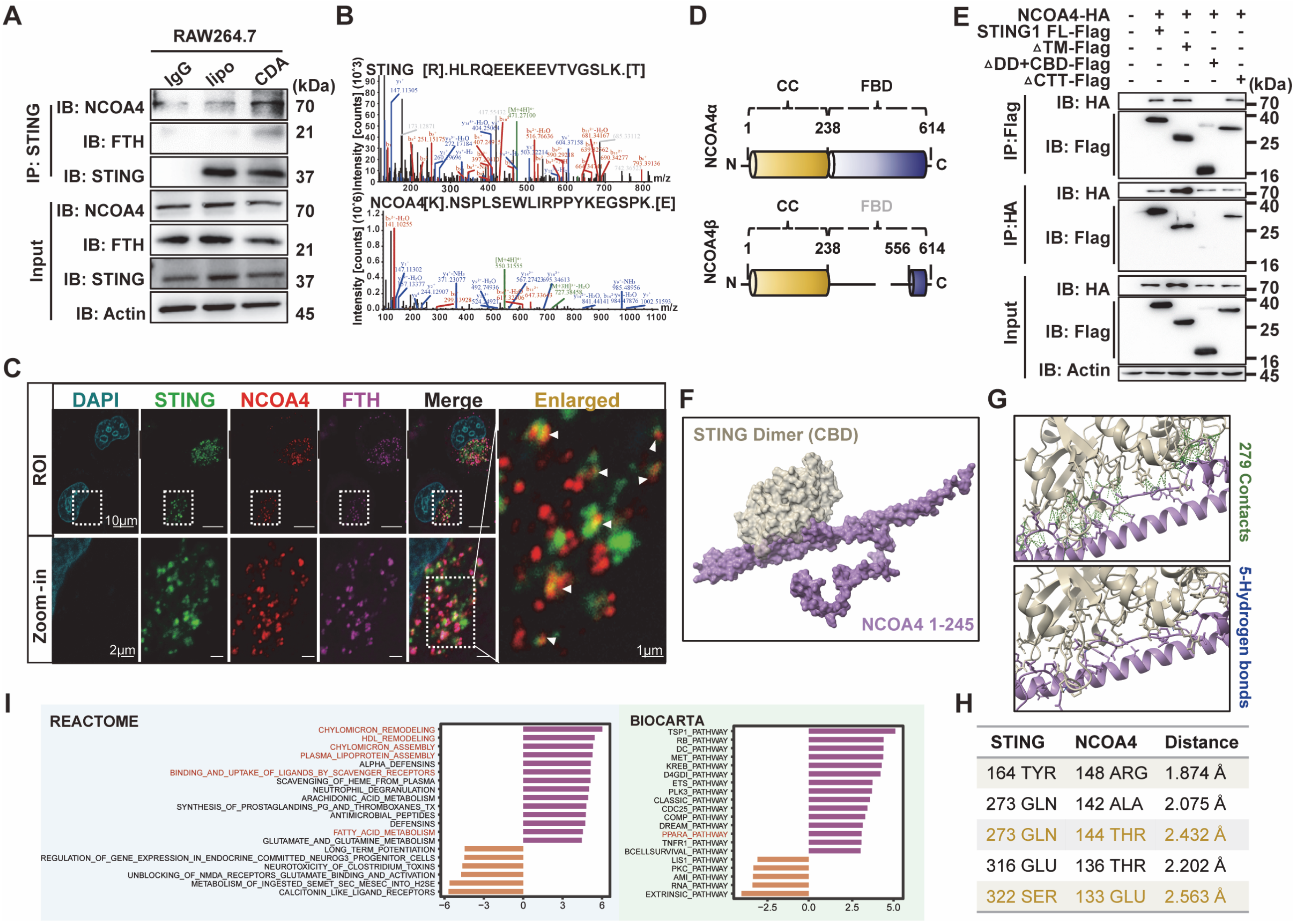

Figure S5. Interaction between STING and NCOA4. (**A**) STING-IP from lysates of RAW264.7 cells stimulated with CDA were immunoblotted to detect of NCOA4 and FTH proteins. (**B**) Representative peptide fragment of mass spectrum of anti-STING in PBMCs from health control and patients with sepsis. (**C**) Representative images of co-localization with STING (green), NCOA4 (red), and FTH (purple) fluorescent proteins was captured by laser scanning confocal microscopy. Enlarged images showed the confocal microscopy images of STING and NCOA4. (**D**) Diagram showing the two isoforms of NCOA4, known as NCOA4α/β. (**E**) Flag or HA IP from lysates of HEK293T cells overexpressing Flag-tagged *STING1* fragments and HA-tagged NCOA4 were immunoblotted. (**F-H**) 3D diagram of the interaction between STING dimer and NCOA4 (1-245 amino acid). The contact interface (green dash), H-bond (blue dash), and hydrogen bond sites are displayed. (**I**) Differential pathways enriched in WT and *Sting1*$^{-/-}$ mice after CLP model by GSVA. P-values were calculated by two-sided moderated t-tests using limma. Immunoblots data are representative of at least three independent experiments with similar results. CDA: c-di-AMP; MS: mass spectrometry; TM: transmembrane; CBD: CDN-binding domain; DD: dimerization domain; CTT: cytoplasmic-terminal tail; CC: coiled-coil domain; FBD: ferritin-binding domain; FL: full-length.

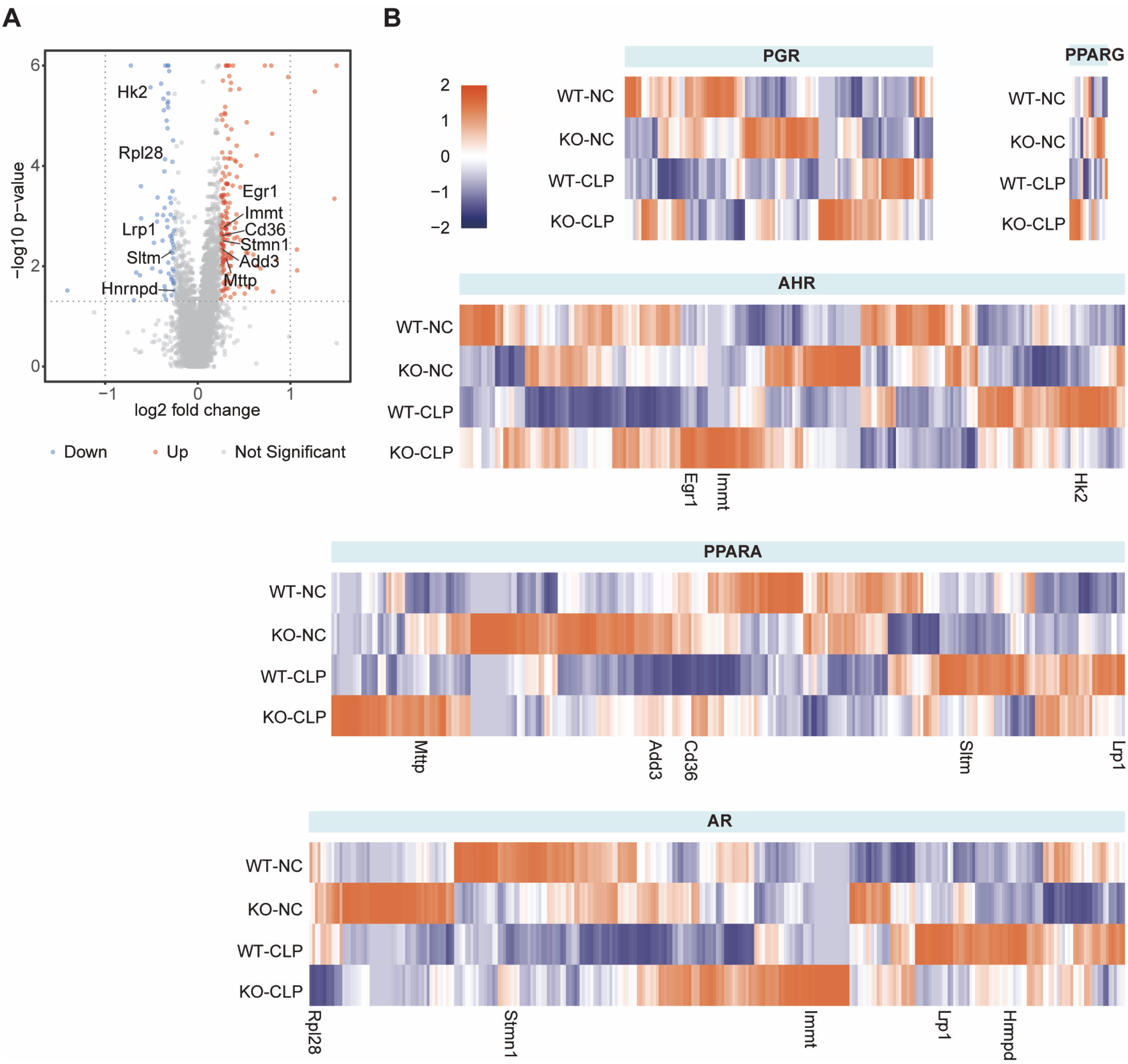

Figure S6. Expression of downstream target genes of transcription factors interacting with NCOA4. (**A**) Volcano plot illustrates the fold differences of all genes in macrophages between *Sting1*$^{-/-}$ and WT mice subjected with CLP model. Horizonal dashed line indicates the p-value cut-off ($P < 0.05$). Genes up or down regulated were marked in red and blue, respectively. PPAR downstream target genes that are significantly differentially expressed were highlighted. (**B**) Heatmap shows the relative expression levels of PGR, AHR, PPARA, PPARG, and AR downstream target genes in macrophages in WT or *Sting1*$^{-/-}$ mice subjected with or without CLP model. DEGs between *Sting1*$^{-/-}$ and WT mice subjected to CLP model were highlighted. GSVA: gene set variation analysis; GSEA: gene set enrichment analysis; TFs: transcription factors.

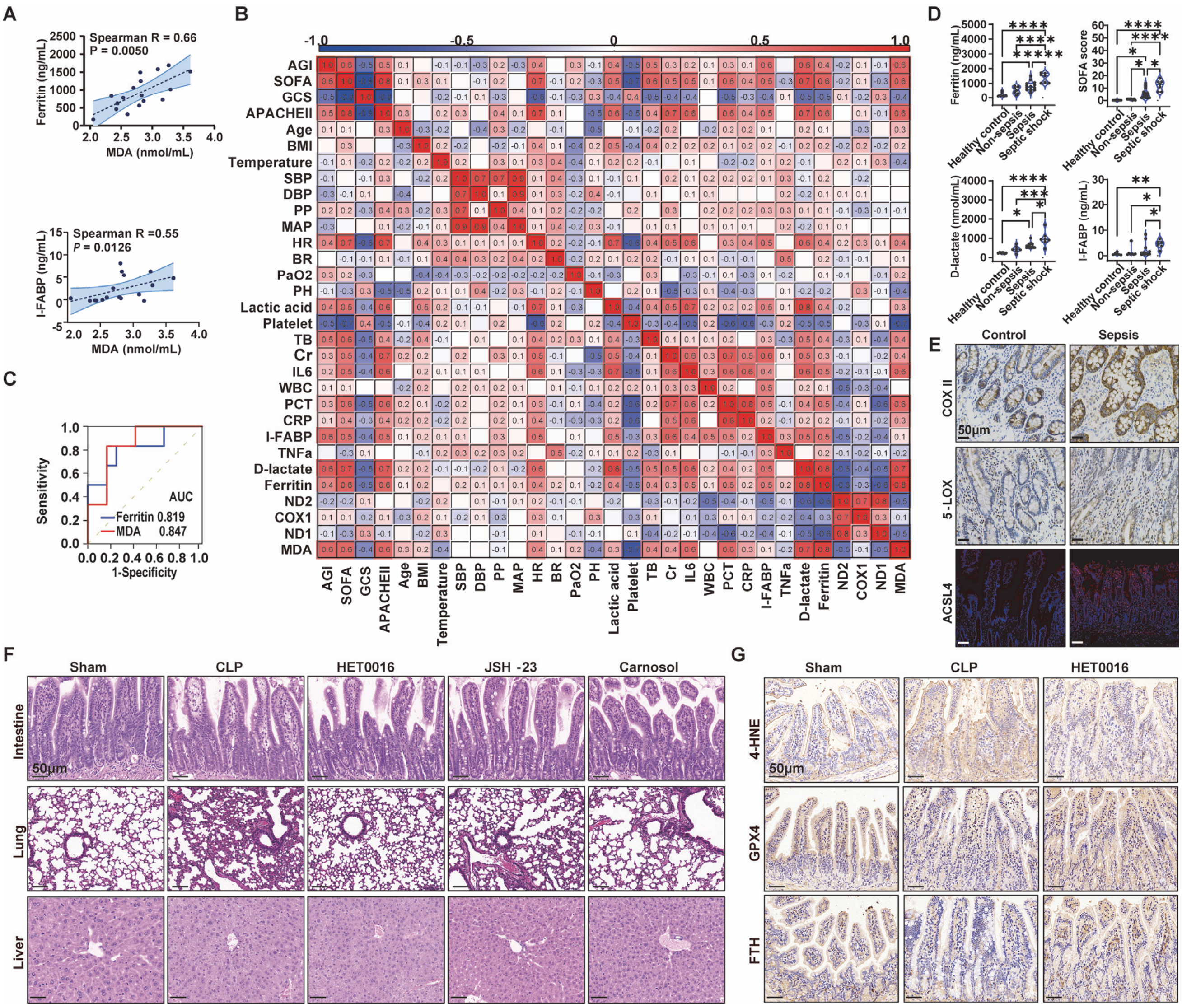

Figure S7. Ferroptosis plays an essential role in lethal sepsis which can be rescued by HET0016. (**A**) Spearman correlation analysis for plasma MDA and ferritin or intestinal fatty acid-binding protein (I-FABP) in all patients (n = 28). (**B**) Heatmap of Spearman's correlation coefficient among the disease severity score, laboratory testing, and ferroptosis-related biomarkers. (**C**) Receiver operating characteristic (ROC) analysis was constructed to evaluate area under the curve (AUC) used to quantify mortality prediction. (**D**) Patients were divided into three groups according to the definition of sepsis and sepsis shock. Violin plots of ferritin, D-lactate, I-FABP, and SOFA score in these four groups are shown (n = 28). (**E**) Representative immunohistochemical or immunofluorescent images of the indicated proteins in intestinal mucosa tissues from patients with sepsis. The scale bar represents 50 μm. (**F**) Representative histopathological section images of intestine, lung, and liver at 24 h after CLP. The scale bar represents 50 μm. (**G**) Representative immunohistochemical images of 4-HNE, GPX4 and FTH in intestinal tissues of CLP models at 24 h after CLP. The scale bar represents 50 μm. Data are shown as mean ± SD, and analysis by one-way ANOVA test (**D**). HC: health control; PBMCs: peripheral blood mononuclear cells; MODS: multiple organ dysfunction syndromes; MDA: malondialdehyde; FTH: ferritin heavy chain; GPX4: glutathione peroxidase 4; 4-HNE: 4-hydroxynonenal. *P <0.05, ** P <0.005, *** P < 0.0005, **** P < 0.0001.

Supplementary Table 1. The 170 ferroptosis related genes were included in this study.

**Increase labile iron**		**Decrease labile iron**		**Antioxidation**	**Peroxidation**	**Promote ferroptosis**		**Inhibit ferroptosis**	
ABCB7	SFXN1	ABCB6	HP	ABCC1	ACACA	ABCC1	IFSP1	ACO1	SLC3A2
ABCB8	SFXN2	ABCG2	IBA57	AIFM2	ACSF2	ACACA	IREB2	ACSL3	SLC40a1
ACO1	SFXN3	ADGB	ISCU	AKR1C6	ACSL3	ACSF2	KEAP1	AIFM2	SLC7A11
ATM	SFXN4	ADHFE1	MB	ALDH3A1	ACSL4	ACSL4	LONP1	AKR1C6	SREBF1
ATP6AP1	SFXN5	ALAS1	MMS19	CARS	AKR1C6	ALOX12	LPCAT3	ALDH3A1	
ATP7A	SLC11A1	ALAS2	NDRG1	CBS	ALOX12	ALOX15	MDM2	CBS	
BMP2	SLC11A2	BACH1	NFS1	DHODH	ALOX15	ALOX5	MDM4	CDKN1A	
BMP5	SLC22A17	BDH2	NFU1	FAR1	ALOX5	ALOX8	NCOA4	CISD1	
BMP6	SLC25A28	CAR9	NGB	FECH	ALOX8	ALOXE3	NOX1	CISD2	
CCDC115	SLC25A37	CDX2	PCBP1	GCLC	ALOXE3	ATM	NOX4	DHODH	
CD163	SLC25A39	CIAO1	PCBP2	GPX4	CS	ATP5G3	PEBP1	FBXL5	
CUBN	SLC39A14	CISD1	PINK1	GSS	CYBB	BAP1	PGD	FECH	
ERFE	SLC39A8	CISD2	QSOX1	IFSP1	CYP1A2	BECN1	RPL8	FTH1	
FLVCR2	SLC46A1	CP	RBCK1	KEAP1	CYP2D	CARS	SAT1	FTL1	
GDF15	SLC48A1	CPOX	SIRT1	MT1	CYP2E1	CS	SLC11A2	GCLC	
GNPAT	SMAD4	FBXL5	SLC40A1	NFE2L2	DPP4	CYBB	SLC1A5	GPX4	
HAMP	STEAP1	FDXR	TET2	NQO1	FADS2	CYP1A2	SLC46A1	GSS	
HJV	STEAP2	FECH	TMPRSS6	SESN2	FDFT1	CYP2D	SLC48A1	ISCU	
HPX	STEAP3	FLVCR1	UROD	SLC3A2	LPCAT3	CYP2E1	SQLE	MT1	
IREB2	STEAP4	FTH1	USF1	SLC7A11	NOX1	DPP4	TFRC	MTF1	
ITLN1	TFR2	FTL1	USF2		NOX4	EMC2	TRF	NFE2L2	
LCN2	TFRC	FTMT			PEBP1	FADS2	VDAC2	NFS1	
LRP1	TLR4	FXN			SAT1	FAR1	VDAC3	NQO1	
LTF	TRF	GLRX3			SQLE	FDFT1	WWTR1	OTUB1	
MTF1	TRP53	GMFG			XDH	FLVCR2	XDH	PCBP1	
MYC	UBE4A	HEPH				G6PDX	YAP	PCBP2	
OTUD1	WDR45	HERC2				GLS2	ZEB1	PPARA	
PICALM		HFE				GOT1		SESN2	

Supplementary Table 2. Demographic and clinical characteristics of patients and healthy control.

	**Healthy control**	**Non-Sepsis**	**Sepsis***	**Sepsis shock**
**Numbers**	6	7	14	7
**Age, years, Mean (SD)**	33.17 (3.545)	48.71 (20.990)	50.00 (17.746)	56.71 (11.940)
**Gender, male (%)**	50.0	71.4	78.6	71.4
**SOFA score, Mean (SD)**	N/A	0.71 (0.488)	5.42 (3.605)	12.50 (4.550)
**APACHE II score, Mean (SD)**	N/A	5.29 (4.152)	12.36 (7.510)	20.29 (6.075)
**Primary Disease, n (%)**				
Trauma	N/A	2 (28.57%)	6 (42.86%)	3 (42.86%)
Surgical complication	N/A	2 (28.57%)	6 (42.86%)	3 (42.86%)
Spontaneous gastrointestinal perforation	N/A	2 (28.57%)	1 (7.14%)	1 (14.28%)
Suppurative appendicitis	N/A	1 (14.29%)	1 (7.14%)	N/A
**In hospital motality, (%)**	0	0	7.14%	100.0%

* Patients were all diagnosed with bacterial sepsis.
