# Supplementary material for: The interaction between STING and NCOA4 exacerbates lethal sepsis by orchestrating ferroptosis and inflammatory responses in macrophages
Source: Cell Death Dis. 2022 Jul 28;13(7):653. doi: 10.1038/s41419-022-05115-x (PMC9334269; doi:10.1038/s41419-022-05115-x)

Figure2. J

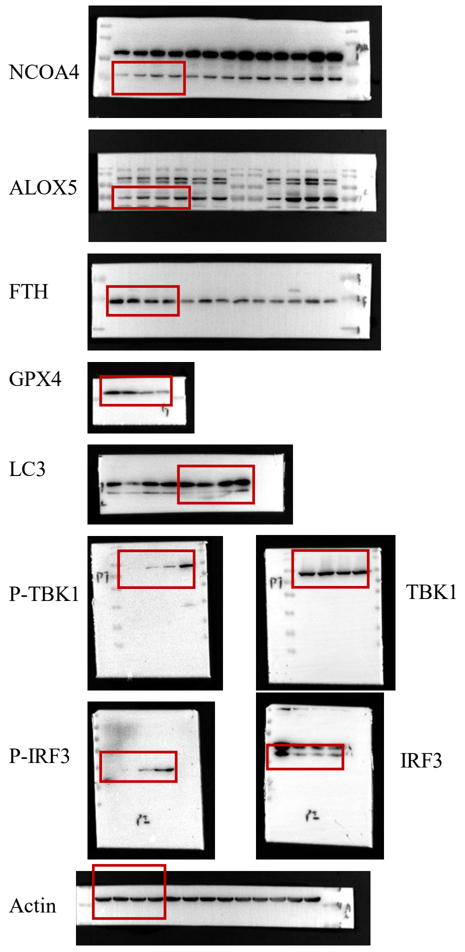

Figure2. K

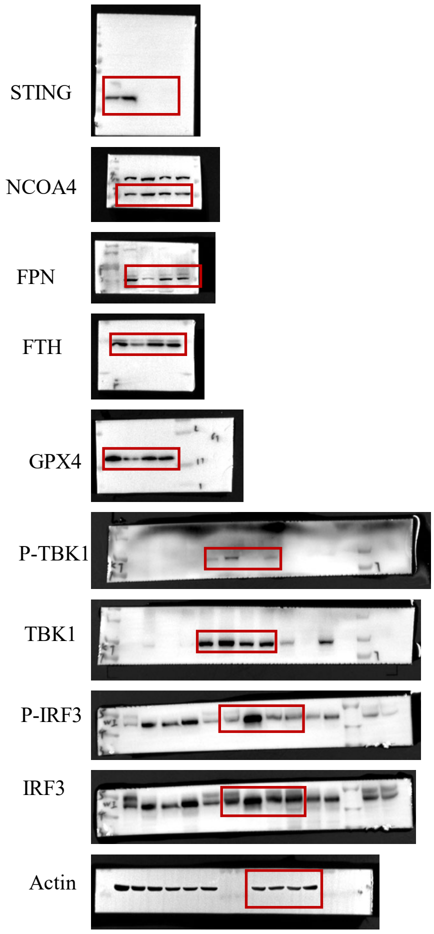

Figure 2.O

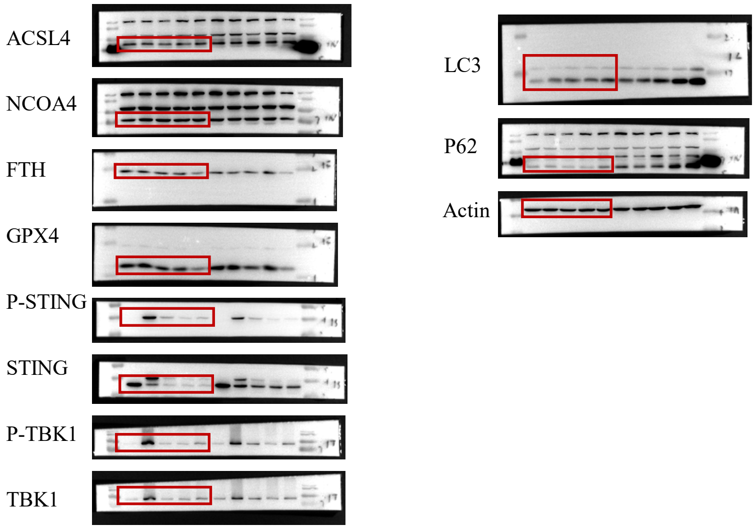

Figure 3. B

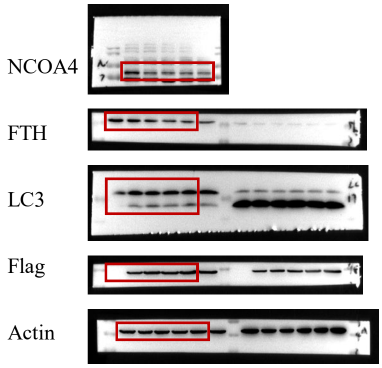

Figure 3. C

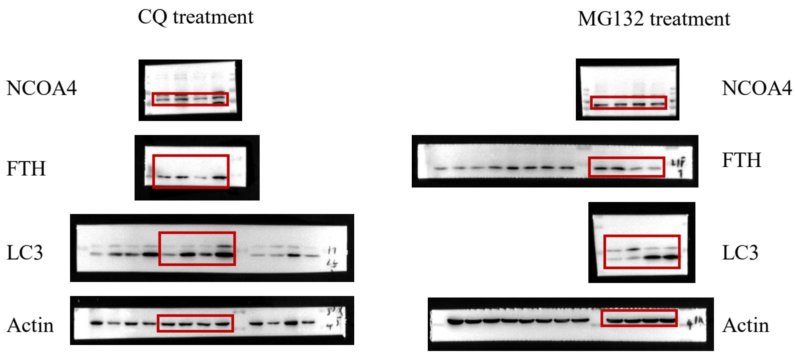

Figure 3. D

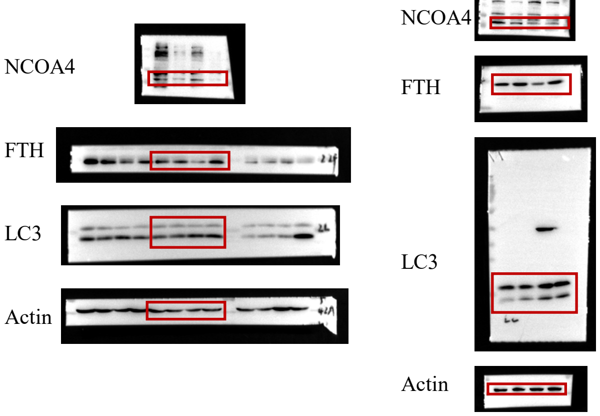

Figure 3. G

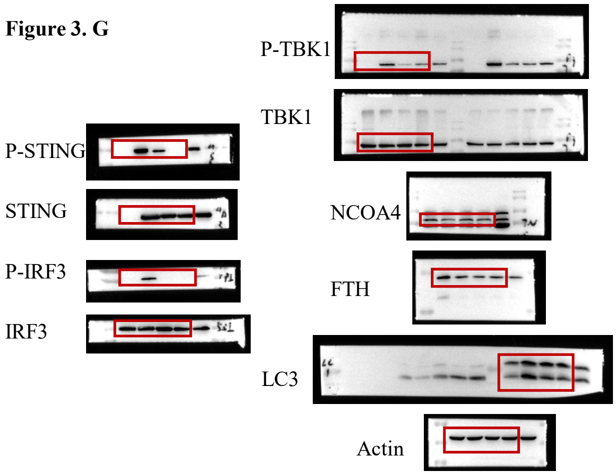

Figure 3. H

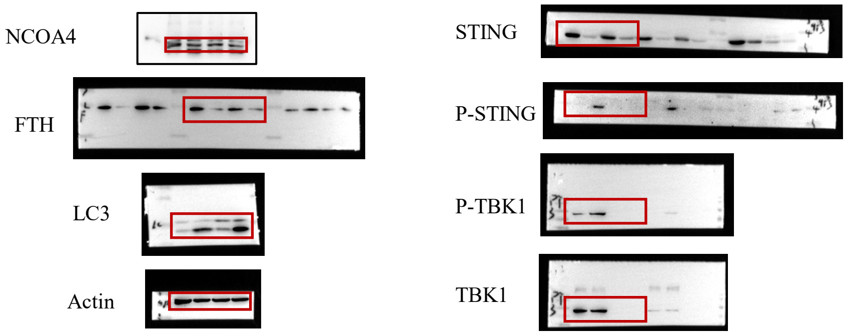

Figure 4. A

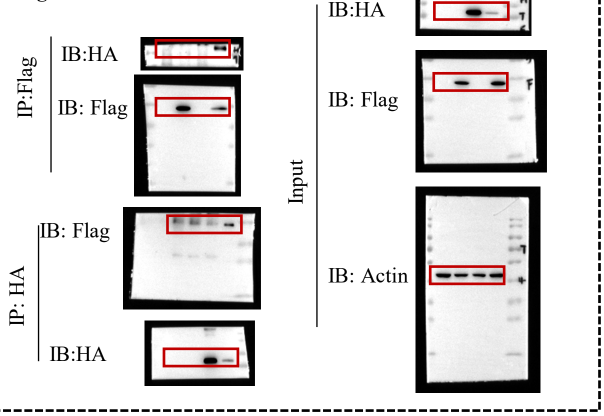

Figure 4. B

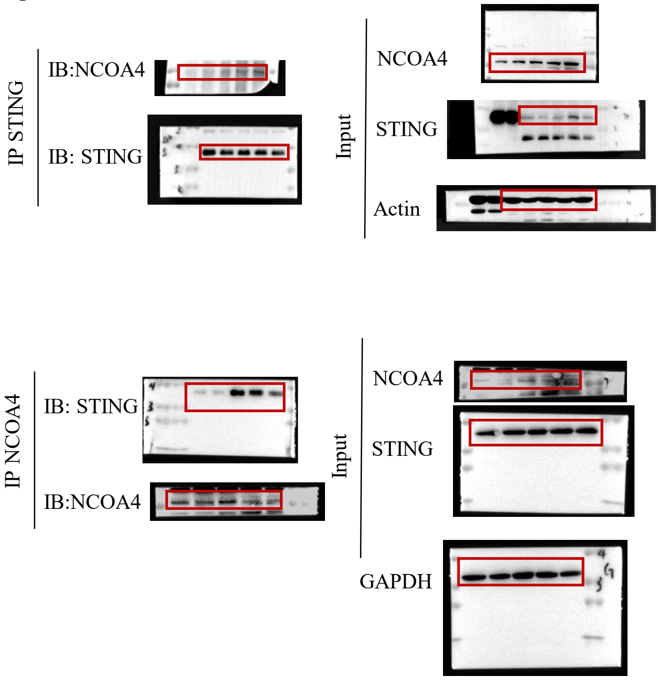

Figure 4. D

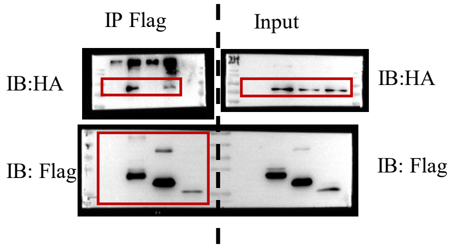

Figure 4. E

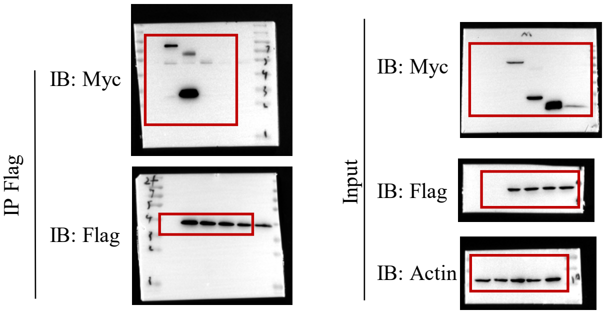

Figure 4. F

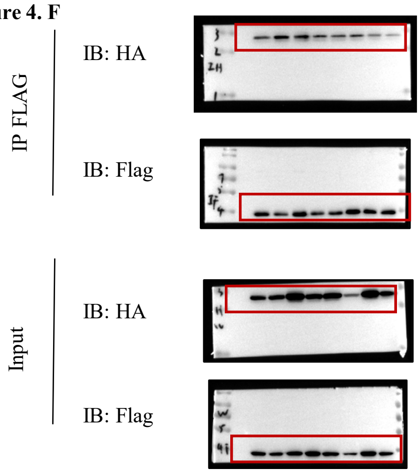

Figure 5. D

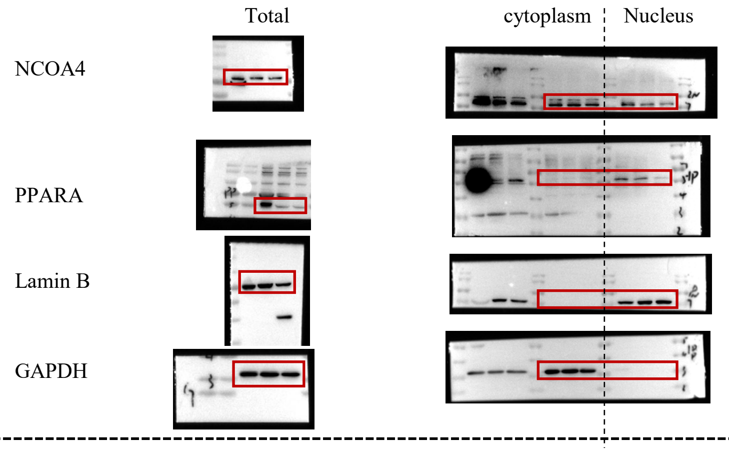

Figure 5. I

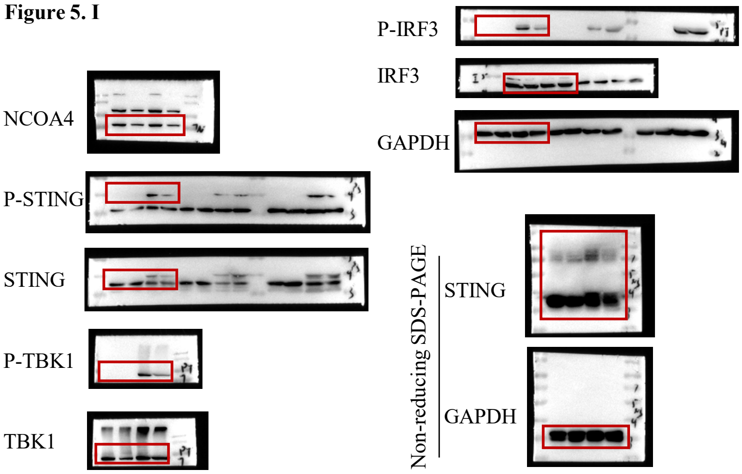

Figure 5. J

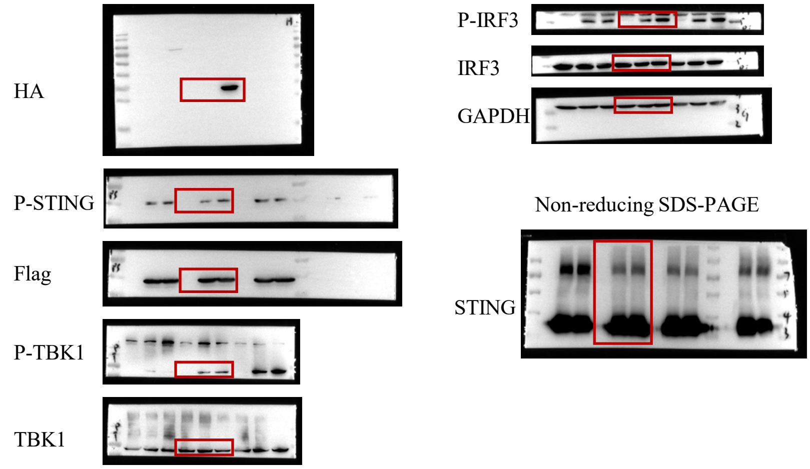

Figure 6. F

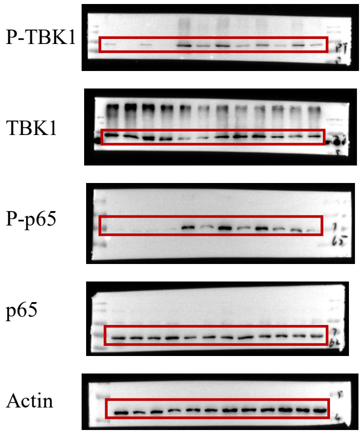

Figure. S3 A

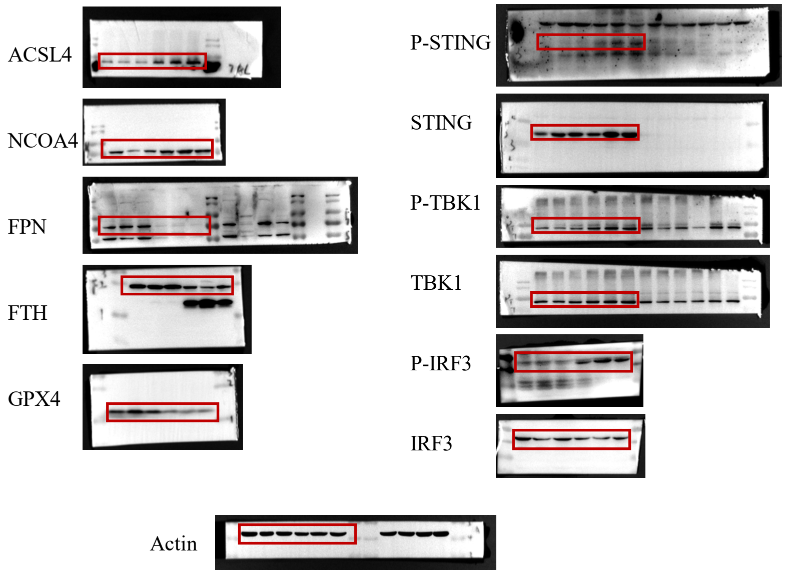

ACSL4
NCOA4
FPN
FTH
GPX4
P-STING
STING
P-TBK1
TBK1
P-IRF3
IRF3
Actin

Fig. S4 b

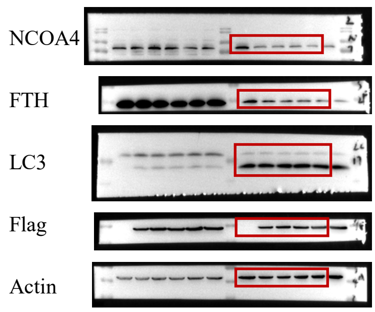

Fig. S4 c

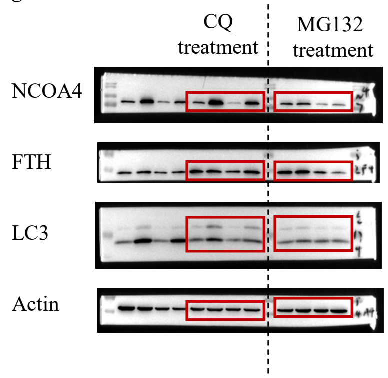

Fig. S4 d

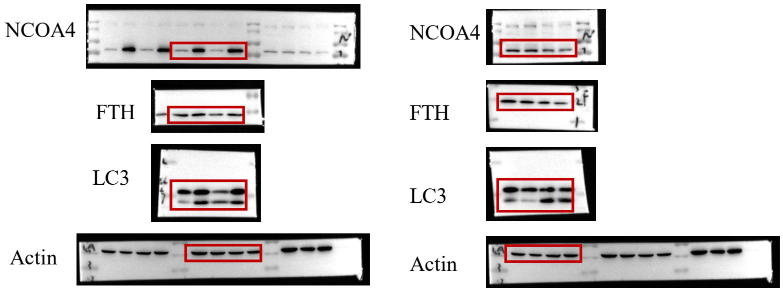

Fig. S4 f

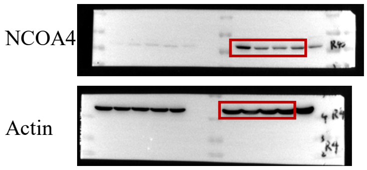

Fig. S4 g

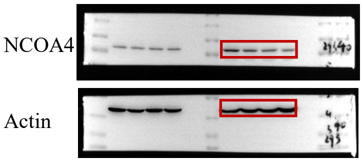

Figure. S5 a

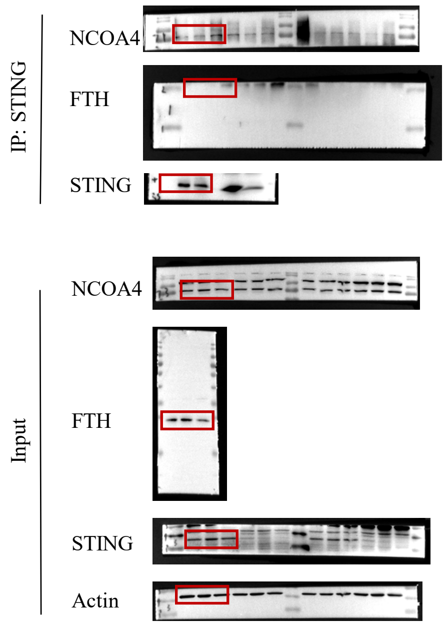

Figure S5. E

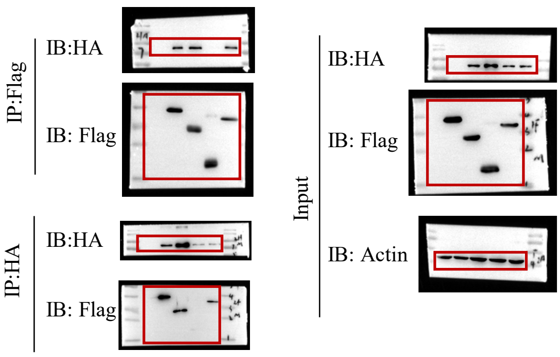

Supplement: Supplementary file 3 — This file contains western blot full scans [file 41419_2022_5115_MOESM3_ESM.pdf]
